# The sialyl-glycolipid stage-specific embryonic antigen 4 marks a subpopulation of chemotherapy-resistant breast cancer cells with mesenchymal features

**DOI:** 10.1186/s13058-015-0652-6

**Published:** 2015-11-25

**Authors:** Andrea Aloia, Evgeniya Petrova, Stefan Tomiuk, Ute Bissels, Olivier Déas, Massimo Saini, Franziska Maria Zickgraf, Steve Wagner, Saskia Spaich, Marc Sütterlin, Andreas Schneeweiss, Manuel Reitberger, Silvia Rüberg, Bernhard Gerstmayer, David Agorku, Sebastian Knöbel, Annalisa Terranegra, Monica Falleni, Laura Soldati, Martin Ronald Sprick, Andreas Trumpp, Jean-Gabriel Judde, Andreas Bosio, Stefano Cairo, Olaf Hardt

**Affiliations:** Miltenyi Biotec GmbH, Friedrich-Ebert-Strasse 68, 51429 Bergisch Gladbach, Germany; XenTech SAS, 4 rue Pierre Fontaine, 91000 Evry, France; Present address: Department of Virology, Pasteur Institute, 25-28 Rue du Docteur Roux, 75015 Paris, France; Heidelberg Institute for Stem Cell Technology and Experimental Medicine (HI-STEM) gGmbH, Im Neuenheimer Feld 280, 69120 Heidelberg, Germany; Division of Stem Cells and Cancer, German Cancer Research Center (DKFZ), Im Neuenheimer Feld 280, 69120 Heidelberg, Germany; Frauenklinik, University Medical Centre Mannheim, Theodor-Kutzer-Ufer 1-3, 68167 Mannheim, Germany; National Center for Tumor Diseases, University Hospital Heidelberg, Im Neuenheimer Feld 460, 69120 Heidelberg, Germany; Sidra Medical and Research Center, PO Box 26999, Doha, Qatar; Department of Health Sciences, University of Milan, Via Festa del Perdono 7, 20122 Milan, Italy; German Cancer Consortium, Im Neuenheimer Feld 280, 69120 Heidelberg, Germany; University of Ferrara, LTTA Centre,Department of Morphology, Surgery and Experimental Medicine, Via Fossato di Mortara 70, 44121 Ferrara, Italy

## Abstract

**Introduction:**

Chemotherapy resistance resulting in incomplete pathologic response is associated with high risk of metastasis and early relapse in breast cancer. The aim of this study was to identify and evaluate biomarkers of treatment-resistant tumor cells.

**Methods:**

We performed a cell surface marker screen in triple-negative breast cancer patient-derived xenograft models treated with standard care genotoxic chemotherapy. Global expression profiling was used to further characterize the identified treatment-resistant subpopulations.

**Results:**

High expression of sialyl-glycolipid stage-specific embryonic antigen 4 (SSEA4) was found in residual tumor cells surviving chemotherapy and in samples from metastatic patients who relapsed after neoadjuvant chemotherapy. Gene and microRNA (miRNA) expression profiling linked SSEA4 positivity with a mesenchymal phenotype and a deregulation of drug resistance pathways. Functional assays demonstrated a direct link between epithelial–mesenchymal transition (EMT) and SSEA4 expression. Interestingly, SSEA4 expression, EMT, and drug resistance seemed to be regulated posttranscriptionally. Finally, high expression of CMP-*N*-acetylneuraminate-β-galactosamide-α-2,3-sialyltransferase 2 (ST3GAL2), the rate-limiting enzyme of SSEA4 synthesis, was found to be associated with poor clinical outcome in breast and ovarian cancer patients treated with chemotherapy.

**Conclusions:**

In this study, we identified SSEA4 as highly expressed in a subpopulation of tumor cells resistant to multiple commonly used chemotherapy drugs, as well as ST3GAL2, the rate-limiting enzyme of SSEA4 synthesis, as a predictive marker of poor outcome for breast and ovarian cancer patients undergoing chemotherapy. Both biomarkers and additionally identified regulatory miRNAs may be used to further understand chemoresistance, to stratify patient groups in order to avoid ineffective and painful therapies, and to develop alternative treatment regimens for breast cancer patients.

**Electronic supplementary material:**

The online version of this article (doi:10.1186/s13058-015-0652-6) contains supplementary material, which is available to authorized users.

## Introduction

Breast cancer is a heterogeneous disease at molecular and cellular levels. Several subtypes of breast cancer can be defined, depending on molecular marker expression [1], and disease management is currently tailored according to the molecular characteristics of each subtype. Triple-negative breast cancer (TNBC) and the luminal B subtype are characterized as very aggressive and associated with high risk of early relapse and metastasis [2, 3]. Owing to the lack of defined molecular targets for TNBCs and due to the high proliferative rate of TNBCs and luminal B tumors, chemotherapy remains a first-choice therapeutic option for these two subtypes.

In the neoadjuvant setting, the standard options for TNBC are a combination of doxorubicin/cyclophosphamide (AC) or 5-fluorouracil, epirubicin, and cyclophosphamide followed or not by taxane-containing regimens, or a combination of cyclophosphamide, methotrexate, and 5-fluorouracil [4]. The majority of TNBC patients respond initially to neoadjuvant chemotherapy treatment, but only about 20 % reach a pathological complete response, whereas most patients either have lower de novo sensitivity to chemotherapy or develop resistance to chemotherapy [5]. Residual cancer cells persist and initiate tumor recurrence and metastasis within 3 years after chemotherapy in about 40 % of patients [5]. Hence, the development of predictive tests for chemotherapy resistance represents an urgent need that would aid in therapy decision-making.

Many studies have been reported that provide data about the molecular basis of human breast cancers. However, the high degree of heterogeneity within the tumor, as well as the varying response to chemotherapy of cellular subclones, makes the interpretation of these molecular profiles difficult [6]. In particular, if biomarkers correlating with treatment outcome are expressed only in subpopulations of tumor cells, the analysis of bulk tumor material might lack sensitivity. Furthermore, tumor cell subpopulations can change their phenotype and gene expression profile to escape chemotherapy by means of epithelial–mesenchymal transition (EMT), upregulation of multidrug resistance transporters, and modulation of key signaling pathways [7, 8]. Thus, it is crucial to analyze marker expression patterns at different time points during chemotherapy, when the tumor cells are either developing drug resistance or de novo resistant subpopulations are selected.

In the present study, we performed a flow cytometry–based screen of cell surface markers in patient-derived xenografts (PDXs) of TNBC tumor samples during AC chemotherapy. PDXs exhibit morphology, molecular characteristics, and drug response profiles similar to those of the original patient tumors [9] and thereby represent a reliable model and a reproducible source for human tumor cells needed for detailed analyses of tumor cell subpopulations over time [9, 10].

We observed that a high percentage of residual tumor cells surviving chemotherapy at surgical ablation express the sialyl-glycolipid stage-specific embryonic antigen 4 (SSEA4). Molecular profiling revealed mesenchymal traits as well as upregulation of genes involved in multidrug resistance in SSEA4-positive compared with SSEA4-negative tumor cell subpopulations. Elevated expression of CMP-*N*-acetylneuraminate-β-galactosamide-α-2,3-sialyltransferase 2 (ST3GAL2), the enzyme catalyzing the last step of SSEA4 synthesis, is associated with poor prognosis in breast cancers treated with chemotherapy. This predictive value was also confirmed in a cohort of ovarian carcinoma. Thus, we propose SSEA4 as a novel marker for EMT-associated chemotherapy resistance and *ST3GAL2* expression as a predictive marker for tumor resistance to chemotherapy.

## Methods

A detailed description of materials and methods can be found in additional file 1.

### Primary tissue material and xenotransplantation

Human breast cancer xenografts (HBCx) were established from patient’s primary tumor surgical specimens by grafting tumor fragments into the interscapular fat pad and maintained through in vivo passages as previously described [9]. All experiments were performed in accordance with French legislation concerning the protection of laboratory animals and in accordance with a currently valid license issued by the French Ministry for Agriculture and Fisheries for experiments on vertebrate animals. The ethics committee was organized according to the pertinent French legislation and was approved by the French Ministry of Research under number CE 51.

Primary serous ovarian carcinoma cell lines were established by transplantation of primary tumor specimen or tumor cells directly isolated from ascites or pleural effusion samples. Human tumors were injected intraperitoneally into NOD.Cg-*Prkdc*^*scid*^*Il2rg*^*tm1Wjl*^ mice. Engrafted first passage xenografts were dissociated into single cells and maintained under serum-free culture conditions. Animal care and all procedures were carried out according to German legal regulations and were previously approved by the governmental review board of the state of Baden-Wuerttemberg (Regierungspräsidium Karlsruhe authorization number G17/12).

This study was performed with human tissue samples obtained from patients admitted to the University Clinic Mannheim Department of Gynecology. The study was approved by the ethics committee of the University of Heidelberg-Mannheim (case number 2011-380N-MA) and conducted in accordance with the Declaration of Helsinki. Written informed consent was obtained from all patients. In addition, primary patient samples of clear cell renal cell carcinoma (RCC) were obtained from the Department of Health Sciences at the University of Milan. All samples were collected according to the regulations for the use of primary material according to “doc. web n. 1878276” (Pubblicato sulla Gazzetta Ufficiale n. 72; 26 Mar 2012).

### Cell lines used

The epithelial breast cell line MCF 10A was purchased from the American Type Culture Collection (ATCC® CRL-10317™; ATCC, Manassas, VA, USA). The HBCx-17 and HBCx-39 cell lines were primary cells derived for the respective HBCx tumors at XenTech SAS (Evry, France). The OC-12, OC-14, OC-15, OC-18, OC-19, and OC-20 cell lines were primary cells derived for the respective ovarian cancer xenograft tumors at HI-STEM gGmbH (Heidelberg, Germany).

### Chemotherapeutic treatment

Doxorubicin (ADRIBLASTINA® RD; Pfizer, New York, NY, USA) and cyclophosphamide (ENDOXAN®; Baxter Healthcare, Deerfield, IL, USA) solutions were administered on the same day via intraperitoneal injection at a dose of 2 mg/kg (doxorubicin) and 100 mg/kg (cyclophosphamide). To obtain a complete response for models HBCx-17 and HBCx-6, the same dose of AC chemotherapy was applied a second time, 3 weeks after the first injection. AC chemotherapy was applied to 68 mice of tumor graft model HBCx-17, 32 mice of HBCx-10, 35 mice of HBCx-6, and 30 mice of HBCx-14 model, not including the control group.

### Flow cytometry–based analysis

Tumor tissue was dissociated into a single-cell suspension using the human Tumor Dissociation Kit in combination with the gentleMACS Octo Dissociator (both from Miltenyi Biotec, Bergisch Gladbach, Germany) according to the manufacturer’s instructions. Cells were stained with the indicated antibodies (Additional file 2: Table S1) according to the manufacturer’s instructions and analyzed using the MACSQuant™ Analyzer (Miltenyi Biotec) (Additional file 3: Figure S1). In the cases of SSEA4, TRA-1-60, and TRA-1-81, recombinant antibodies were available and were used because of their superior characteristics [11, 12]. The specificity of all recombinant antibodies was validated and compared with conventional clones. In the case of SSEA4, identical specificity for antibodies derived from clone MC-813-70 and clone REA101 was proven by cross- blocking experiments, which showed that either antibody specifically blocks binding of the alternative one, suggesting that both antibodies bind the same epitope on the target structure SSEA4 (Additional file 4: Figure S2).

### Isolation of SSEA4-positive and SSEA4-negative tumor cell subpopulations

SSEA4-positive and SSEA4-negative tumor cell subpopulations were isolated by magnetic activated cell sorting (MACS® Technology; Miltenyi Biotec). After dissociation and depletion of mouse cells using the Mouse Cell Depletion Kit (Miltenyi Biotec), the cells were labeled with SSEA4-phycoerythrin (Miltenyi Biotec) followed by anti-phycoerythrin MicroBeads (Miltenyi Biotec) and separated using MS and LD columns (Miltenyi Biotec). For microarray analysis, cells were pelleted and lysed in QIAzol® (QIAGEN, Hilden, Germany).

### EMT induction

To induce EMT, the epithelial breast cell line MCF 10A was treated with transforming growth factor (TGF)-β1 (Miltenyi Biotec) at concentrations of 10 and 20 ng/ml. EMT markers such as epithelial cell adhesion molecule (EpCAM), E-cadherin, vimentin, and fibronectin served as indicators of EMT induction efficiency.

### Microarray hybridization and data analysis

Messenger RNA (mRNA) and microRNA (miRNA) expression profiling was performed using Agilent microarrays (Agilent Technologies, Santa Clara, CA, USA) according to the manufacturer’s instructions.

The mRNA and miRNA data discussed in this publication have been deposited in the National Center for Biotechnology Information Gene Expression Omnibus (GEO) [13, 14] and are accessible under GEO series accession number [GEO:GSE57705] (http://www.ncbi.nlm.nih.gov/geo/query/acc.cgi?acc=GSE57705).

### Clinical data analysis

To evaluate the prognostic value of candidate genes, the publicly available Kaplan-Meier Plotter (http://kmplot.com/analysis/) was used. The Kaplan-Meier survival plots of the two patient cohorts were compared using log-rank test with hazard ratios (HRs) and 95 % confidence intervals [15, 16].

### In vitro cytotoxicity assays

Cells were seeded in 96-well flat-bottom plates. After 2 days at 37 °C and 5 % CO_2_, the cells were treated with different standard chemotherapy drugs. To obtain a dose–response curve, each drug was tested at serial concentrations. Cell viability was analyzed 72 h after addition of drugs using the CellTiter-Glo® Luminescent Cell Viability Assay Kit (Promega, Madison, WI, USA) according to the manufacturer’s instructions. Luciferase activity was measured on a luminometer (PerkinElmer® EnVision™; PerkinElmer, Waltham, MA, USA).

## Results

### The expression of 23 cell surface markers in breast cancer PDXs is affected by chemotherapy

**Fig. 1:**
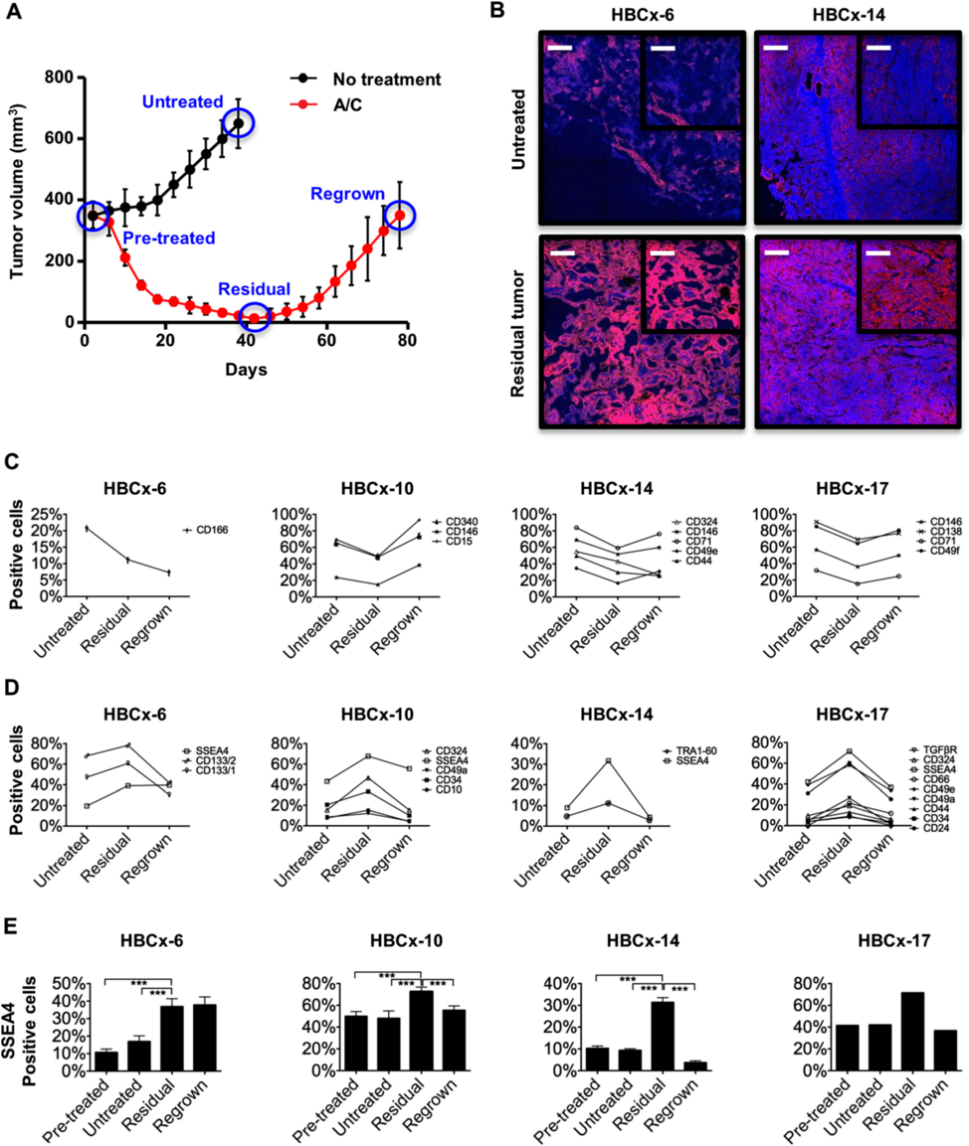
Identification of differentially regulated cell surface markers during chemotherapeutic treatment. **a** Study design for chemotherapy treatment of xenograft tumors, blue circles around graphed points represent time points of analysis . **b** Immunohistochemical analysis of sialyl-glycolipid stage-specific embryonic antigen 4 (SSEA4) expression (*red*, SSEA4; *blue*, 4′,6-diamidino-2-phenylindole). **c**, **d** The expression patterns of cell surface markers in xenograft tumors from four individual breast cancer patients were analyzed by multiparametric flow cytometry during the course of treatment. **e** The expression of SSEA4 during chemotherapeutic treatment of xenograft tumors was analyzed at four time points (*n* = 8). ***p < 0.001; scale bars = 300 μm for larger images and 100 μm for insets. *A/C* doxorubicin/cyclophosphamide, *HBCx* human breast cancer xenograft, *TGFβR* transforming growth factor β receptor

An antibody screening based on a library of 45 antibodies directed against surface epitopes including published stem cell and/or cancer stem cell markers (Additional file 2: Table S1) was performed to identify novel biomarkers for breast cancer cell subpopulations resistant to chemotherapeutic treatment. For the initial screening, 50–100 xenografted mice were used for each of four independent TNBC PDXs [9]. When tumor volumes reached 150–350 mm^3^ (pretreatment stage), mice were treated with an AC combination according to the standard of care. After tumor shrinking to volumes of 14–63 mm^3^, the nodules were removed (residual tumor stage). Tumors from untreated mice were removed (untreated stage) to serve as direct controls. A group of animals with residual tumors were kept until the disease recurred (regrowth stage) (Fig. 1a). All tumors removed at any time point were dissociated and analyzed by flow cytometry. No significant differences concerning marker expression were found between the pretreatment and untreated stages, excluding size-dependent marker regulation, whereas the expression of 10 markers decreased (Fig. 1c) and the expression of 13 markers increased (Fig. 1d) during chemotherapy. Eighty-seven percent (20 of 23) of these markers returned to an expression level similar to the untreated stage upon tumor regrowth (Fig. 1c and d). CD44 and CD133, which have been described to correlate with a cancer stem cell and drug resistance phenotype [17], showed enrichment in only one of the four models. This is possibly due to the tumor heterogeneity that characterizes patients’ tumors, which was here well recapitulated by the use of PDX models (Fig. 1d). However, we identified a distinct subpopulation of tumor cells expressing SSEA4 that was strongly enriched during chemotherapeutic treatment in all tumor models analyzed. Independent replicates of three tumor models confirmed significant enrichment in the number of SSEA4-positive cells in residual tumors upon AC treatment in all analyzed tumors (*p* < 0.001, *n* = 8 for each tumor model) (Fig. 1e). This result was further confirmed by immunohistochemistry (Fig. 1b). Similarly to other markers, the amount of SSEA4-positive cells decreased back to pretreatment levels in three of four tumor models after the treatment was stopped.

To further address the correlation between CD24, CD44, and SSEA4 expression, we performed containing of these markers on residual tumor nodules after AC chemotherapy and on untreated tumors of three independent models (Additional file 5: Figure S3). We did not observe a significant enrichment of the CD24^low^/CD44^high^ subpopulation in any of the models. In model HBCx-6, there was a subpopulation of SSEA4^+^/CD44^+^ cells that was enriched by about twofold; however, the SSEA4^+^/CD44^−^ fraction was enriched to the same degree. In models HBCx-10 and HBCx-14, CD44 and SSEA4 expression was observed on separate subpopulations. We concluded that SSEA4 expression is an indicator of treatment-resistant cells that overcomes the heterogeneity observed for the (cancer) stem cell markers in the tumor models used in this study.

### SSEA4 is a marker for de novo resistance to chemotherapy treatment

In one PDX model, we observed tumors with variable sensitivity to the AC treatment and noticed that the amount of SSEA4-positive cells correlated with these differences. Nineteen tumors showed reduced sensitivity to AC treatment (tumor volume >100 mm^3^ as maximal regression), and one tumor did not respond to the treatment at all (no tumor shrinkage during treatment). The percentages of SSEA4-positive cells were 36.8 % in sensitive tumors, 42.2 % in tumors with reduced sensitivity, and 98.5 % in the fully resistant tumor (data not shown). Therefore, we compared tumors from PDX models that are sensitive (*n* = 6) or de novo resistant (*n* = 4) to AC treatment. Three of four resistant tumor models showed higher percentages of SSEA4-positive cells than the six sensitive tumors (Additional file 6: Figure S4).

**Fig. 2:**
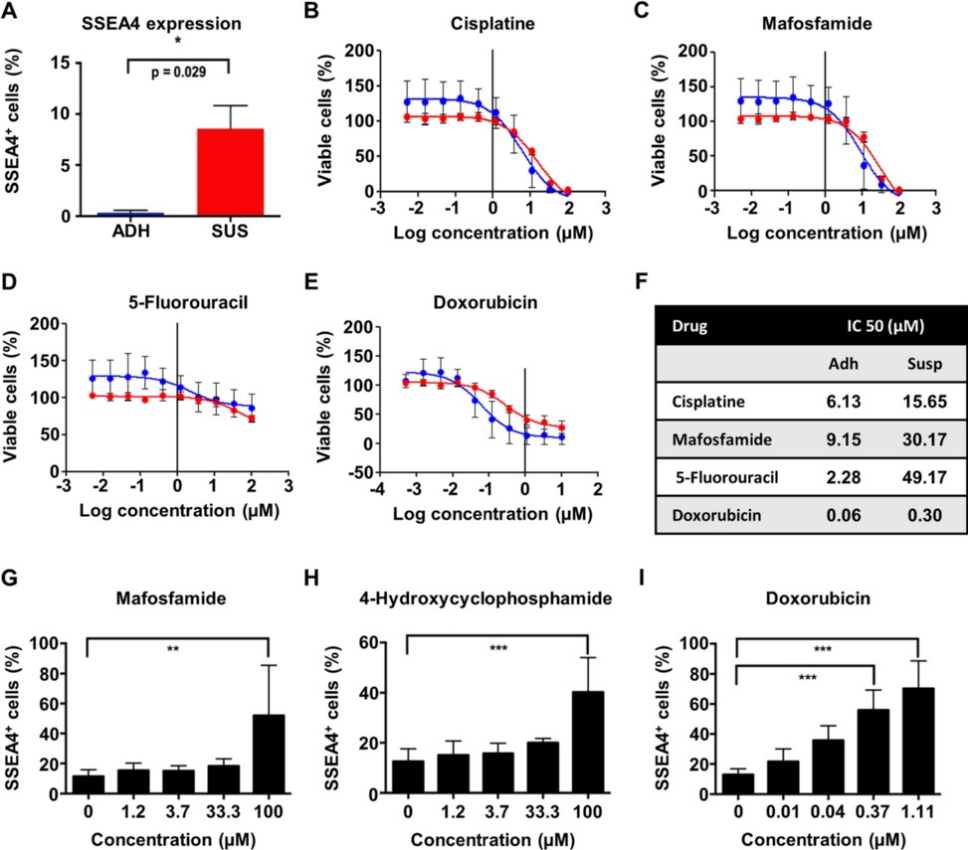
Expression of sialyl-glycolipid stage-specific embryonic antigen 4 (SSEA4) during chemotherapeutic treatment of breast cancer cells in vitro*.* Human breast cancer xenograft (HBCx)-17 cells containing different amounts of SSEA4-positive cells were treated with chemotherapeutic drugs in vitro (**a**). The half-maximal inhibitory concentration (IC_50_) values (*n* = 3) for the commonly used drugs cisplatin (**b**), mafosfamide (**c**), 5-fluorouracil (**d**), and doxorubicin (Adriamycin/ADRIBLASTINA® RD) (**e**) were measured. The suspension cells showed higher IC_50_ values, indicating an increased resistance to those drugs (**f**). To directly examine the phenotype of cells surviving the treatments, a purely adherent cell line derived from model HBCx-17 was treated with increasing concentrations of mafosfamide (**g**), 4-hydroxycyclophosphamide (**h**), or doxorubicin (**i**) (*n* = 4). **p < 0.01, ***p < 0.001. *ADH* adherent culture, *SUS* suspension culture

To provide further evidence of resistance to drug toxicity, we established primary PDX-derived ex vivo cell lines from tumors containing different amounts of SSEA4-positive cells and treated them with chemotherapeutic drugs in vitro. One of the cell lines derived from model HBCx-17 showed reproducible growth as an adherent culture and as a suspension culture, with cells growing in suspension showing higher SSEA4 expression than the adherent cells (Fig. 2a). In cytotoxicity assays with seven commonly used drugs (Fig. 2b-f and data not shown), the suspension cells showed higher half-maximal inhibitory concentration values than the adherent cells for the DNA synthesis and transcription inhibitors cisplatin, mafosfamide, 5-fluorouracil, and doxorubicin, indicating an increased resistance, but not for the topoisomerase inhibitors etoposide, topotecan, and irinotecan. To provide a dose-escalating reproduction of our in vivo experiment, we treated an adherent cell line derived from model HBCx-17, containing about 15 % SSEA4-positive cells in the steady state, and analyzed the phenotype of the cells surviving the treatment upon administration of increasing drug dosages. In every case, the surviving population showed a significantly higher fraction of SSEA4-positive cells (Fig. 2g-i), while the absolute number of SSEA4-positive cells was not significantly increased.

Next, we evaluated possible differences in the tumorigenic capacity of SSEA4-positive and SSEA4-negative subpopulations in model HBCx-14, which shows about 10 % SSEA4-positive tumor cells when growing without any treatment and a significant upregulation under genotoxic stress. To do so, 10^5^ freshly dissociated SSEA4-positive or SSEA4-negative cells were injected into two groups of eight mice each. Although a trend toward a faster tumor initiation was observed when we grafted the SSEA4-positive tumor subpopulation, both fractions were able to generate tumors, indicating that the positive fraction only has an initial growth advantage (Additional file 7: Figure S5a). Furthermore, we analyzed the expression of SSEA4 in tumors that arose from both subpopulations. Whereas the fraction of SSEA4-expressing cells in tumors that originated from the SSEA4-negative subpopulation recovered to around 5 %, the one from tumors that originated from the SSEA4-positive subpopulation was reduced to about 10 % following the growth phase in vivo (Additional file 7: Figure S5b). The recovery of the original SSEA4-positive versus SSEA4-negative ratio was also reflected by the observation that when freshly dissociated SSEA4-positive and SSEA4-negative cells were placed in culture separately, they restored the original positive versus negative ratio within approximately 6 days (data not shown), which correlated well with the observation of SSEA4 downregulation during disease relapse after chemotherapy.

### SSEA4 expression is found in metastatic cells that survived genotoxic chemotherapy

**Fig. 3:**
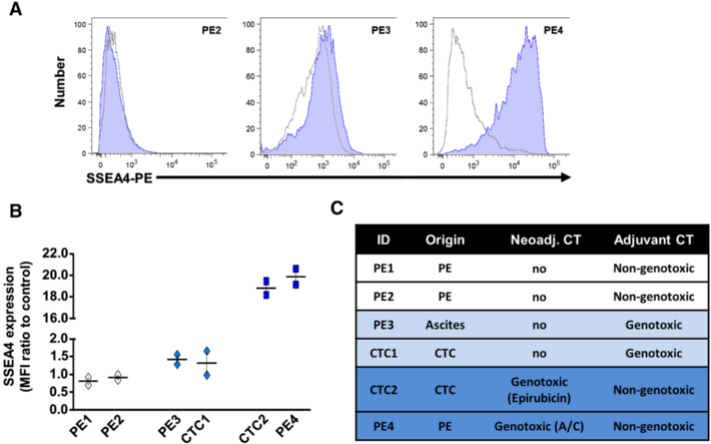
Sialyl-glycolipid stage-specific embryonic antigen 4 (SSEA4) expression is found in metastatic cells that survived genotoxic chemotherapy. **a** The surface expression of SSEA4 on primary cells from patient-matched xenografts (passages 1–3) was evaluated. Each xenograft was stained with an isotype control (*gray histogram*) or with an SSEA4 antibody (*blue histogram*). **b** Quantification of SSEA4 expression on the cell surface of six xenografts obtained from metastatic pleural effusions (PE) or circulating tumor cells (CTCs) from M1-stage metastatic breast cancer patients. The median fluorescence intensity (MFI) ratio is calculated as the MFI of SSEA4-stained cells divided by the MFI of the isotype control-stained cells. **c** Schematic overview of each metastatic breast cancer patient’s treatment characteristics. *CT* chemotherapy)

Local or metastatic breast cancer relapse may occur many years after surgery, despite a short-term response to neoadjuvant chemotherapy [18] or a failure of adjuvant chemotherapy. To investigate SSEA4 expression in metastatic relapse, we conducted a retrospective case history study using PDXs derived from metastatic specimens. These models were derived from confirmed M1-stage patients through collection of liquid biopsies, either peripheral circulating tumor cells (CTCs) or infiltrating CTCs obtained from serous effusions [19]. Robust SSEA4 expression was exclusively found in PDXs whose donors were previously treated with specific neoadjuvant chemotherapy formulations, including the AC combination and other genotoxic drugs with analogous modes of action, such as epirubicin (Fig. 3). Conversely, metastatic patients who were administered taxols (paclitaxel, docetaxel) or antimetabolites (capecitabine, 5-fluorouracil) as adjuvant therapy, but who had no genotoxic treatment before sample collection (Fig. 3 and Additional file 8: Table S2 for patient history), displayed little or no expression of SSEA4 on their matched PDXs (Fig. 3).

### Molecular analysis of SSEA4-positive and SSEA4-negative subpopulations

**Fig. 4:**
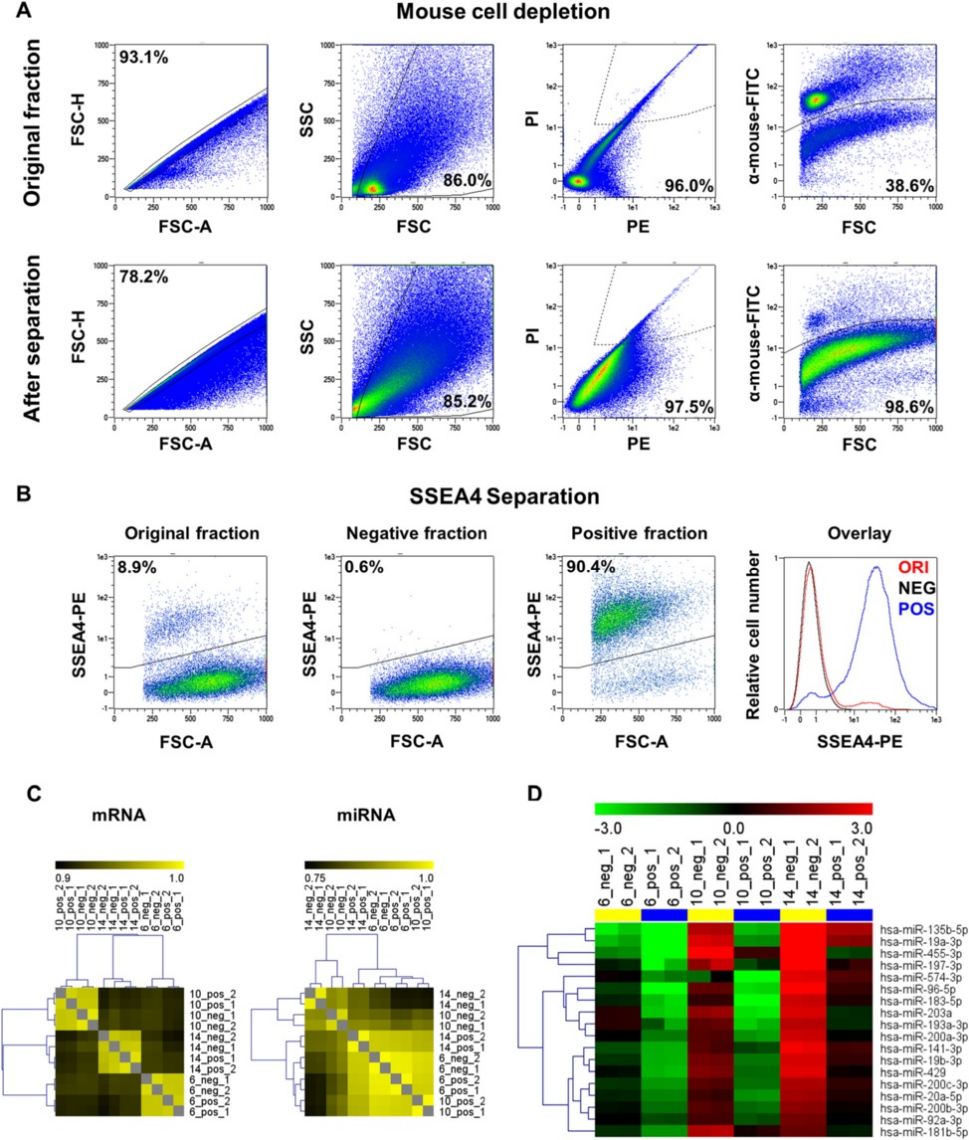
Isolation and molecular analysis of sialyl-glycolipid stage-specific embryonic antigen 4 (SSEA4)-positive and SSEA4-negative tumor subpopulations. **a** and **b** Flow cytometric analysis of cells before and after cell sorting. **c** Correlation matrices showing the relationship of mRNA- and miRNA-based gene expression profiles in all experiments. Correlation coefficients are indicated by their color from 0.9 (*black*) to 1.0 (*yellow*) for mRNA-based and from 0.75 (*black*) to 1.0 (*yellow*) for miRNA-based clustering. **d** Cluster analysis of miRNAs identified as differentially expressed by discriminatory gene analysis in combination with a paired *t* test (*p* < 0.05) of the SSEA4-positive versus SSEA4-negative tumor subpopulation. Resulting genes were grouped by similarities in gene expression patterns using hierarchical clustering (Pearson correlation, average linkage). Levels of log_2_-transformed expression ratios are indicated from −3 (*green*) to 3 (*red*). *FITC* fluorescein isothiocyanate, *FSC* forward scatter *FSC-A* forward scatter area, *FSC-H* forward scatter height, *miRNA* microRNA, *mRNA* messenger RNA, *PE* pleural effusions, *PI* propidium iodide, *SSC* side scatter

We performed microarray-based global mRNA and miRNA expression profiling on SSEA4-positive and SSEA4-negative cells using three independent TNBC models: HBCx-6 (*n* = 7 tumors), HBCx-10 (*n* = 5 tumors), and HBCx-14 (*n* = 9 tumors). To avoid a bias by cells of murine origin [20], all mouse cells were depleted after tumor dissociation (Fig. 4a) and SSEA4-positive cells were labeled and magnetically separated from the negative fraction (Fig. 4b) before expression profiling.

Transcripts with significantly increased (240 genes, *p* < 0.05, ≥1.5-fold, Additional file 9: Table S3) or decreased (182 genes, *p* < 0.05, greater than or equal to −1.5-fold, Additional file 9: Table S3) expression in SSEA4-positive compared with SSEA4-negative cells were subjected to a term enrichment analysis based on Gene Ontology categories [21]. In the SSEA4-positive fractions, we found a strong overrepresentation of genes linked to the TGF-β and epidermal growth factor signaling pathways as well as genes involved in cell adhesion and migration and in regulation of apoptosis, proliferation, and differentiation (Additional file 9: Table S3). Among the genes upregulated across all tumor models, seven functional groups involved in cellular import and export, response to toxins and oxidative stress were significantly enriched (Additional file 9: Table S3). In particular, members of the solute carrier (SLC) and multidrug resistance ATP-binding cassette transporter families were significantly upregulated (Additional file 10: Figure S6B).

Also, among the differentially expressed transcripts, we identified a substantial number of genes involved in EMT. Epithelial markers such as cytokeratin 19*, CLDN1*, *CLDN3*, and *CLDN4* showed lower expression in SSEA4-positive cells, whereas mesenchymal indicators such as fibronectin, vitronectin, *ZEB1*, and *ZEB2* were upregulated (Additional file 10: Figure S6 A). In contrast, published stem cell markers were not consistently regulated among the SSEA4-positive and SSEA4-negative cell fractions (Additional file 10: Figure S6 C). As SSEA4 is a glycolipid epitope, its expression cannot be monitored directly by transcriptome profiling. SSEA3, the direct precursor of SSEA4, showed no increased signal intensity in SSEA4-positive cells when measured with anti-SSEA3 antibody in all analyzed models (data not shown). This indicates that SSEA4 enrichment regulation may be due to increased SSEA3-to-SSEA4 conversion or to increased SSEA4 catabolism in SSEA4-negative cells. However, no difference was observed in the expression of genes coding for enzymes involved in degradation of SSEA4 or of ST3GAL2, the enzyme catalyzing this final step of SSEA4 synthesis [22]. In expression analysis of the miRNA data set, we identified 166 miRNAs more than twofold overrepresented and 68 miRNAs more than twofold underrepresented in SSEA4-positive versus SSEA4-negative cells among all analyzed tumor models (Additional file 9: Table S3). Hierarchical clustering of the Pearson correlation coefficients of our mRNA and miRNA expression datasets showed a higher correlation of SSEA4-positive versus SSEA4-negative phenotype in the miRNA rather than mRNA data set (Fig. 4c). No miRNAs were significantly upregulated, and 18 miRNAs were significantly downregulated (*p* < 0.05 by paired *t* test), in SSEA4-positive cells (Fig. 4d and Additional file 9: Table S3). These results were independently validated by using a flow cytometry–based 39-plex miRNA assay (Additional file 11: Figure S7) for direct measurement of miRNAs without prior amplification. To determine putative mRNA targets for these candidates, we used six different computational target prediction tools and considered only targets that were predicted by at least two target prediction algorithms. On the basis of this analysis, 5 of the 18 miRNAs downregulated in SSEA4-positive cells (miR-96-5p, miR-200b-3p, miR-200c-3p, miR-429, and miR-92a-3p) are predicted to target *ST3GAL2*, suggesting that SSEA4 expression is regulated posttranscriptionally. Furthermore, 12 of the 18 downregulated miRNAs are known to target key mesenchymal regulator and indicator genes such as *ZEB1*, *ZEB2*, fibronectin 1, *Snail1*, *Snail2*, and *Twist* (Additional file 12: Figure S8 and Additional file 9: Table S3).

### SSEA4 expression is regulated by ST3GAL2

**Fig. 5:**
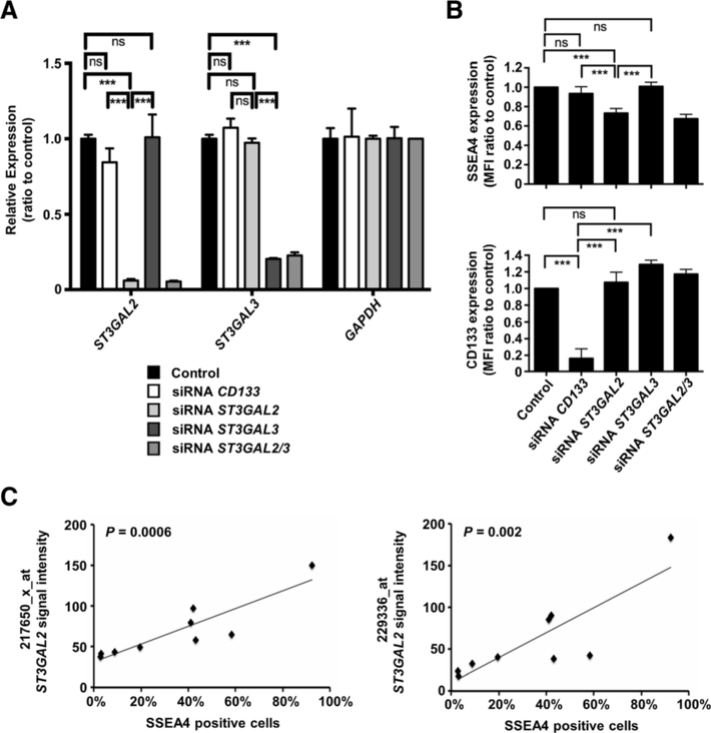
Sialyl-glycolipid stage-specific embryonic antigen 4 (SSEA4) expression is regulated by CMP-*N*-acetylneuraminate-β-galactosamide-α-2,3-sialyltransferase 2 (*ST3GAL2*). **a** Small interfering RNA (siRNA)–mediated knockdown of *ST3GAL2* and *ST3GAL3* significantly reduced the expression of their respective target messenger RNAs (mRNAs), but not the housekeeping gene *GAPDH*, as measured by quantitative real-time polymerase chain reaction. Each bar represents the expression intensity normalized to the Lipofectamine reagent (Life Technologies, Carlsbad, CA, USA)-only control. **b** Knockdown of *ST3GAL2* significantly reduced the expression of SSEA4, whereas targeting of CD133 or the close paralog *ST3GAL3* did not result in a significant change of SSEA4 expression. Knockdown of CD133 expression by the respective siRNA was used as a positive control of direct targeting. Each bar represents the mean fluorescence intensity (MFI) normalized to the Lipofectamine-only control. **c** Expression of *ST3GAL2* mRNA was measured by two Affymetrix® (Affymetrix, Santa Clara, CA, USA) probes, 217650_x_at and 229336_at, in nine tumor models and plotted against the frequency of SSEA4-positive cells as measured by flow cytometry in the respective models, which showed a significant positive correlation. ***p < 0.001; ns = not significant; *n* = 6

To provide functional confirmation of the role of *ST3GAL2* in the regulation of SSEA4 expression in PDX samples, we knocked down *ST3GAL2* by small interfering RNA (siRNA) in HBCx-39 cells, which show high levels of SSEA4 expression, and analyzed the expression of SSEA4. A significant decrease of SSEA4 expression was observed upon *ST3GAL2* expression inhibition (*p* < 0.001, *n* = 6) (Fig. 5a and b). Knockdown of CD133 cell surface expression was used as a positive control for siRNA targeting, and it had no impact on SSEA4 expression. To provide further evidence of the relationship between *ST3GAL2* and SSEA4, we compared *ST3GAL2* mRNA levels with SSEA4 cell surface levels on nine different tumor models, which showed significant positive correlation (*p* < 0.002, Fig. 5c).

### Epithelial–mesenchymal transition induces SSEA4 expression

**Fig. 6:**
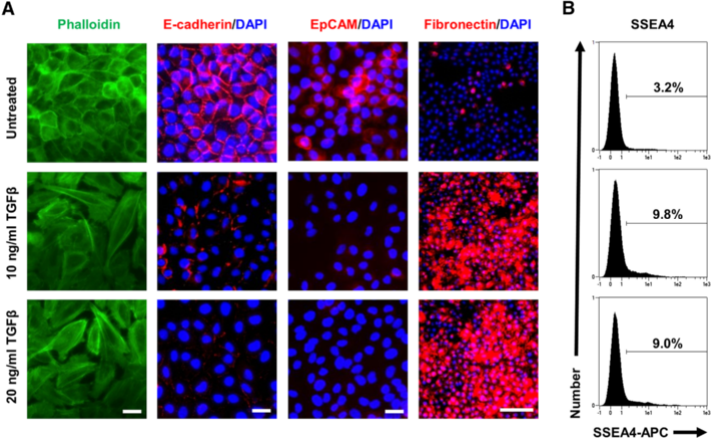
Epithelial–mesenchymal transition (EMT) induces sialyl-glycolipid stage-specific embryonic antigen 4 (SSEA4) expression. **a** The expression of SSEA4 was evaluated upon EMT induction. Upon treatment, almost all cells changed their morphology from an epithelial to an elongated fibroblastic shape. F-actin was stained with phalloidin to visualize the cytoskeleton architecture. The epithelial markers E-cadherin and epithelial cell adhesion molecule (EpCAM) were downregulated, whereas the mesenchymal marker fibronectin was upregulated, upon stimulation. Scale bar = 10 μm (*first three columns at left*) and 200 μm (*right column*). **b** The fraction of SSEA4-positive cells was increased upon EMT induction as evaluated by flow cytometry. *APC* allophycocyanin, *DAPI* 4′,6-diamidino-2-phenylindole, *TGFβ* transforming growth factor β

As EMT has already been correlated with drug resistance [23], we wanted to examine if SSEA4 expression was increased after EMT induction. Upon treatment of the epithelial breast cell line MCF 10A with TGF-β1, almost all cells changed their morphology from an epithelial to an elongated fibroblastic shape (Fig. 6a). As expected [24], epithelial markers such as EpCAM and E-cadherin were downregulated, while mesenchymal markers such as vimentin and fibronectin were upregulated (Fig. 6a and data not shown). Upon EMT induction, the SSEA4-positive fraction increased more than threefold, hence proving a causal correlation between the transition toward a mesenchymal phenotype and SSEA4 expression (Fig. 6b).

### *ST3GAL2* is a highly significant predictive and prognostic marker in breast cancer patients

**Fig. 7:**
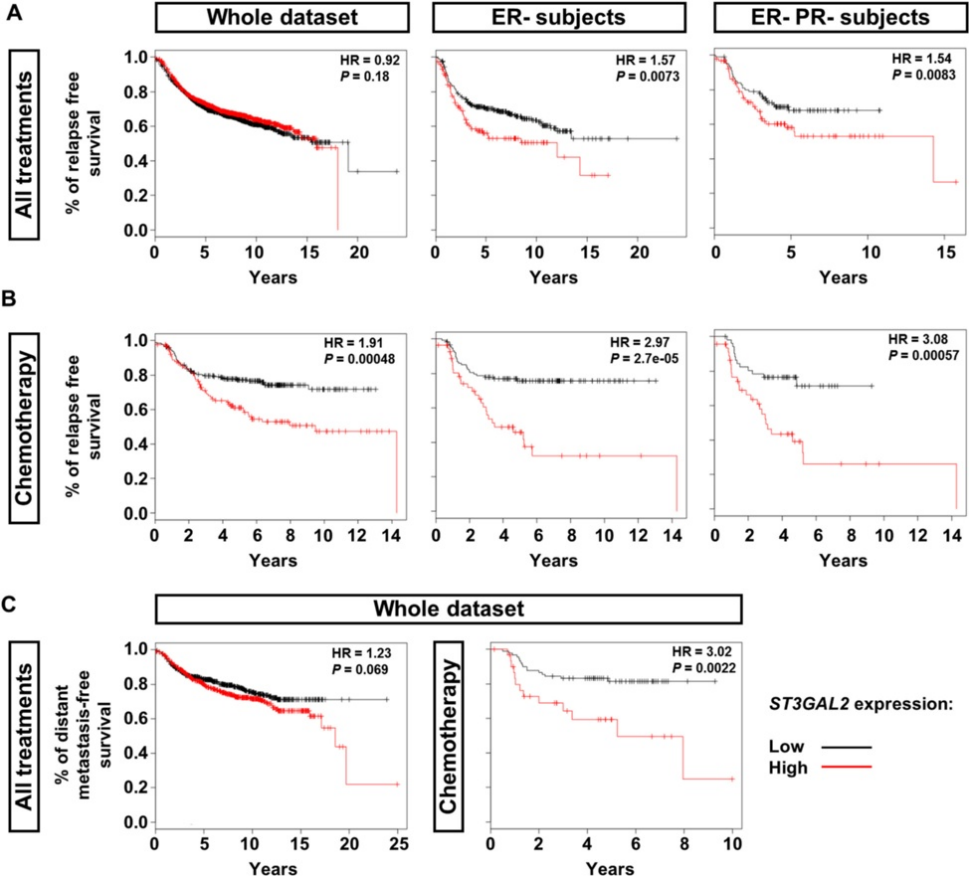
Expression of CMP-*N*-acetylneuraminate-β-galactosamide-α-2,3-sialyltransferase 2 (*ST3GAL2*) correlates with breast cancer patients’ prognosis. The predictive value of *ST3GAL2* was evaluated using a large, publicly available clinical microarray database based on breast tumors from 2977 patients [15]. Estrogen receptor–negative (ER^−^) patients and ER^−^/progesterone receptor –negative (PR^−^) patients displayed highly significant differences (*p* < 0.01) trending toward a poorer prognosis for patients expressing higher levels of *ST3GAL2* (**a**). When we focused on patients treated with chemotherapy, we observed a highly significant reduction of relapse-free survival independent of the tumor subtype [*p* < 0.01, hazard ratio (HR) 1.91] in ER^−^ patients (*p* < 0.01, HR 2.97) and in double-negative patients (*p* < 0.01, HR 3.08) among patients expressing high levels of *ST3GAL2* (**b**). Also, when we applied distant metastasis-free survival as a primary endpoint, we found that patients treated with chemotherapy had a worse prognosis when expressing high levels of *ST3GAL2* (**c**).

Given the functional connection between SSEA4 and *ST3GAL2*, we evaluated the clinical value of *ST3GAL2* in a large, publicly available clinical microarray database of breast tumors from 2977 patients [15]. Highly significant differences (*p* < 0.01) trending toward a poorer outcome for patients expressing higher levels of *ST3GAL2* within the estrogen receptor–negative (ER^−^) or ER^−^/progesterone receptor–negative (PR^−^) subset of patients were found, independent of the treatment (Fig. 7a). When we focused on patients treated with chemotherapy, we observed a highly significant reduction of relapse-free survival independent from the tumor subtype (*p* < 0.01, HR 1.91) in ER^−^ patients (*p* < 0.01, HR 2.97) and in ER^−^/PR^−^ patients (*p* < 0.01, HR 3.08) among patients expressing high levels of *ST3GAL2* (Fig. 7b). When we applied distant metastasis-free survival as an endpoint, we found that patients expressing high levels of *ST3GAL2* had a worse outcome, confirming the involvement of SSEA4-positive cells in metastasis formation (Fig. 7c), as observed in the case of metastasis-derived PDXs.

### SSEA4 and *ST3GAL2* expression predict chemoresistance and are associated with patient outcomes in other carcinomas

**Fig. 8:**
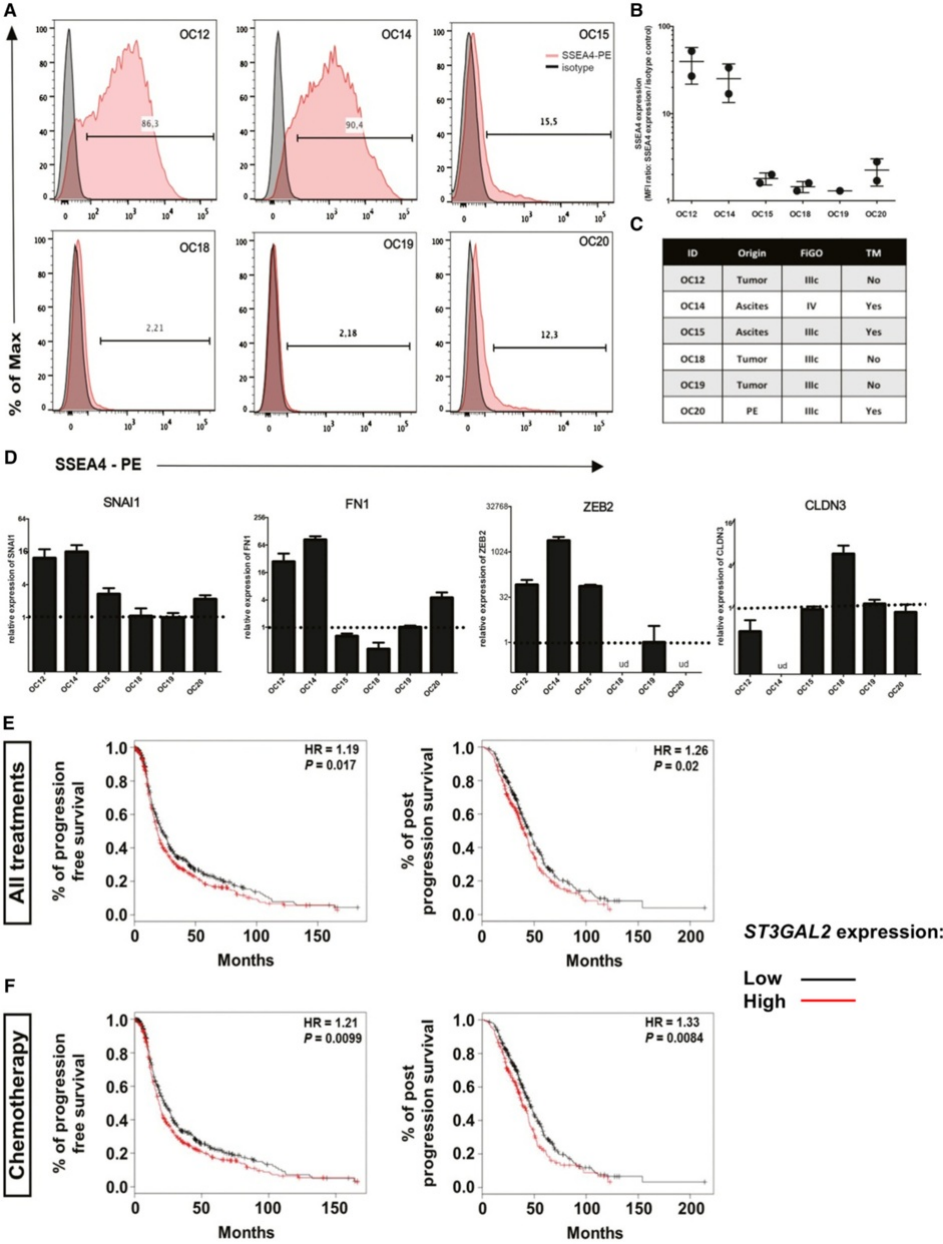
Sialyl-glycolipid stage-specific embryonic antigen 4 (SSEA4) and CMP-*N*-acetylneuraminate-β-galactosamide-α-2,3-sialyltransferase 2 (*ST3GAL2*) expression in ovarian cancer cells correlates with a mesenchymal phenotype and patient prognosis. **a** Flow cytometric analyses of SSEA4-phycoerythrin (*red line*) and respective isotype control (*black line*) on primary cells from patient-matched ovarian cancer cells. Numbers indicate percentage of positive cells compared with the isotype control. **b** Quantification of SSEA4 expression on six patient-matched primary ovarian cancer cell lines expressed as median fluorescence intensity (MFI) ratio. The MFI ratio is calculated as the MFI of SSEA4-stained cells divided by the MFI of the respective isotype control-stained cells. **c** Overview of patient-derived ovarian cancer sample characteristics. *PE* pleural effusion, *TM* treatment, *FIGO* International Federation of Gynecologists and Obstetricians. **d** Quantitative real-time polymerase chain reaction analysis of genes related to mesenchymal (*SNAI1*, *FN1*, *ZEB2*) and epithelial (*CLDN3*) phenotypes in the six patient-matched primary ovarian cancer cell lines. Error bars represent SD (performed in triplicates). *ud* undetected). **e** and **f** *ST3GAL2* expression in ovarian cancer significantly correlates with poor prognosis (**e**), particularly in patients who underwent chemotherapy (**f**). *HR* hazard ratio

To further evaluate SSEA4 expression as a marker of intrinsic tumor cell resistance to chemotherapy, we tested SSEA4 expression in samples from primary clear cell RCC, a tumor entity known to be de novo resistant to chemotherapy in more than 95 % of patients [25] and from late ovarian cancer, an aggressive disease whose treatment relies on chemotherapy as the only therapeutic option [26]. Primary clear cell RCC as well as healthy kidney tissues from the same patients were analyzed for SSEA4 expression. In all of the analyzed patients (*n* = 3), an elevated number of SSEA4-positive cells was observed in the tumor tissue (Additional file 13: Figure S9). Similarly, when primary cells derived from serous ovarian carcinoma specimens were analyzed for expression of SSEA4, three of three samples from tumors that previously received genotoxic treatment showed SSEA4 expression above 10 %, whereas only one primary cell line matched to treatment-naive patients showed SSEA4 expression above 10 % (Fig. 8a–c). Analysis of mesenchyme-specific (*FN1*, *SNAI1*, *ZEB2*) and epithelial (*CLDN3*) genes by quantitative real-time polymerase chain reaction revealed that ovarian cancer primary cells with high SSEA4 expression showed higher expression of mesenchyme-specific genes and lower expression of the epithelium-specific ones (Fig. 8a and b).

In light of these results, we evaluated whether *ST3GAL2* expression could be associated with patient prognosis in ovarian cancer. Using the whole dataset of ovarian tumors from 1464 patients [16], a significant difference trending toward a worse clinical outcome was observed with respect to progression-free survival (*p* < 0.05) as well as postprogression survival (*p* < 0.05) (Fig. 8e). When we assessed patients treated with chemotherapy, the level of significance was also strongly increased for both endpoints (*p* < 0.01) (Fig. 8f).

## Discussion

In the present study, we identified a novel subpopulation of chemotherapy-resistant tumor cells defined by the expression of SSEA4, the highest-order glycosphingolipid (GSL) in the globo series synthetic pathway starting from glucosylceramide. Enrichment of SSEA4-positive cells in residual tumors of all the tumor models used in this study suggests that this pluripotency marker overcomes the heterogeneity shown by many cancer stem markers analyzed (e.g., CD44, CD133, CD117, CD271, ABCG2) [27–29] that were found to be enriched only in single models. A large body of evidence indicates that upregulation of ceramide glycosylation by glucosylceramide synthase, through its ability to increase levels of high-order GSLs, contributes to acquisition of drug resistance in cancer cells [30]. In our experimental model, the fraction of SSEA4-positive cells returned to pretreatment levels in the majority of PDXs upon tumor regrowth, indicating that this subpopulation has no general growth advantage. Indeed, this behavior was proposed for other subpopulations of drug-resistant cells [31]. Accordingly, we did not observe a significant difference in tumorigenic capacity among SSEA4-positive or SSEA4-negative subpopulations. Similar observations have been made in non–small cell lung cancer, where residual tumor cells driving disease relapse after chemotherapy appear to be in an EMT state but do not show any enrichment of cancer stem cell marker–positive cells or enhanced tumor-initiating capacity [23]. Given that SSEA4-positive cell enrichment is a transient event, it is possible that the SSEA4-positive and SSEA4-negative cell balance tends to revert to the original ratio of positive and negative subpopulations observed in the untreated tumor during the latency period between cell injection and macroscopic tumor growth.

Tumors with initially high levels of SSEA4-positive cells seemed to be de novo resistant to chemotherapy. One important question is whether SSEA4 is upregulated during chemotherapy or if preexisting SSEA4-positive cells are selected. The four TNBC PDXs used for the antibody screening had been established from untreated tumors and received a maximum of two cycles of chemotherapy, whereas four to six cycles are administered in the clinic. This might explain why PDXs from metastatic breast cancer, as well as primary cells from ovarian cancers, previously exposed to genotoxic therapy show very high initial percentages of SSEA4-positive cells. It is possible that this difference correlates with reversible or irreversible enrichment of the SSEA4 population.

Molecular analysis suggested a higher correlation of the drug resistance phenotype with the miRNA rather than the mRNA expression profile. Among mRNAs differentially expressed, a significant enrichment of genes involved in cellular import and export, response to toxins, and oxidative stress was observed, pathways that are connected to drug response at least on a global tumor level [32]. This is consistent with the reported role of globo series GSLs in *MDR1* upregulation via the activation of cSrc signaling [33]. One striking observation was the downregulation of epithelial markers in conjunction with the overrepresentation of mesenchymal markers [34] at the mRNA level, and particularly, the concomitant downregulation of their regulatory miRNAs. Sustaining the observation that SSEA4-positive tumor cells show a more mesenchymal phenotype, we also found that induction of EMT enhances SSEA4 expression. This is in concordance with our observation that SSEA4 expression is found in metastatic cells that survived genotoxic chemotherapy. A large body of evidence connects EMT to drug resistance [35, 36] and metastasis [37, 38], providing a mechanistic explanation that might underlie the observed effects in SSEA4-positive tumor cells.

Two of the differentially regulated miRNAs—miR-141 and miR-200a—have been shown to influence resistance to cisplatin and carboplatin in ovarian cancer by controlling the oxidative stress response [39]. We found that SSEA4 expression also correlates with a mesenchymal state and drug resistance in ovarian cancer.

Besides the EMT phenotype, the expression of SSEA4 is also likely regulated by miRNAs among different tumor subpopulations. The direct link between *ST3GAL2* and SSEA4 was proven by siRNA-mediated knockdown of *ST3GAL2* resulting in a significant decrease of SSEA4 expression and by positive correlation between SSEA4 and *ST3GAL2* expression in PDX models. Interestingly, all five miRNAs predicted to target *ST3GAL2* are also directly involved in EMT and drug resistance [39, 40]. Even if further studies clarify whether *ST3GAL2* is directly involved in drug resistance, this regulatory mechanism, in combination with the overexpression of resistance-associated genes, such as transporters of the MDR family, might be the underlying mechanisms linking EMT and drug resistance to SSEA4 expression.

## Conclusions

We have identified SSEA4 to mark a subpopulation of chemotherapy-resistant, mesenchymal breast cancer cells. Furthermore, we have shown that the expression level of *ST3GAL2*, the enzyme catalyzing SSEA4 synthesis, can be used as a marker to predict clinical outcome of breast and ovarian cancer patients, in particular those treated with chemotherapy. SSEA4 and *ST3GAL2* may therefore represent key markers to classify patient groups in order to avoid ineffective and painful therapies and to develop alternative treatment regimens for breast cancer patients.

## Additional files

Additional file 1:
**Supplemental experimental procedures.** Detailed description of materials and methods. (DOCX 62 kb) Additional file 2: Table S1. Antibodies used for the screening approach. Detailed description of used antibodies. (DOCX 46 kb) Additional file 3: Figure S1. Representative gating strategy for flow cytometry–based marker analysis of dissociated xenograft tumor tissue. Tumor tissue was dissociated to obtain a single-cell suspension while preserving cell surface epitopes. The sample was stained for mouse-specific markers to exclude cells of murine origin from the analysis as well as for the screening candidates and analyzed by multiparametric flow cytometry. Doublets were excluded by forward scatter (FSC) area/FSC height gating (**a**); debris was excluded by FSC/side scatter gating (**b**); dead cells were excluded by gating off propidium iodide–positive events (**c**); and mouse cells were excluded by gating on α-mouse-fluorescein isothiocyanate–negative events (**d**). When we screened two samples in parallel, we found that one of the samples was labeled using an ultraviolet dye, allowing for subsequent separation of the events of each sample by gating on the VioBlue channel fluorescence intensity (**e**–**h**). (PNG 736 kb) Additional file 4: Figure S2. Anti-SSEA4 antibodies derived from clone REA101 and MC-813-70 recognize the same epitope. Flow cytometric analysis of an antibody cross-blocking experiment on human induced pluripotent stem cells. Cells were either directly fluorescently labeled using anti-SSEA-4-phycoerythrin conjugates from clone REA101 (**a**) or MC-813-70 (**b**) or after cells had been blocked by preincubation with 100 μg/ml unconjugated antibody of the alternative clone (**a**, **b**). The fluorescent labeling of the REA101-derived anti-SSEA-4-phycoerythrin antibody was strongly diminished by blocking with an excess of MC-813-70 unconjugated antibody (**a**), while unconjugated REA101 caused a complete block of the fluorescent labeling of MC-813-70-derived anti-SSEA-4-phycoerythrin antibody (**b**). These results indicate that both antibodies recognize the same epitope and that the REA101-derived antibody has a higher functional affinity than the one derived from clone MC-813-70. (PNG 107 kb) Additional file 5: Figure S3. Correlation among CD24-, CD44-, and SSEA4-expressing subpopulations. To address the correlation between CD24, CD44, and SSEA4 expression, we performed costaining of these markers on residual tumor nodules after AC chemotherapy and untreated tumors of three independent models: HBCx-6 (**a**), HBCx-10 (**b**), and HBCx-14 (**c**). Regulation of the three markers did not correlate among the treatment cycles. (PNG 1664 kb) Additional file 6: Figure S4. Expression of SSEA4 in tumors responsive or resistant to chemotherapeutic treatment. Tumors responsive (*n* = 6) or resistant (*n* = 4) to AC treatment were analyzed for expression of SSEA4. Three of the four resistant tumor models showed higher percentages of SSEA4-positive cells than all of the six responsive tumors. In two of the resistant tumor models, almost all of the cells expressed SSEA4. (TIFF 61 kb) Additional file 7: Figure S5. Tumor-initiating capacity of the SSEA4-positive and SSEA4-negative subpopulation. (**a**) One hundred thousand freshly dissociated SSEA4-positive or SSEA4-negative cells were injected in two groups of eight mice each. Tumor volume was measured once per week, and the mean volume of both groups was calculated. The significance level (*p* value by *t* test) is indicated above each time point. (**b**) The frequency of SSEA4-expressing cells in the parental tumor model HBCx-14 and in tumors that originated from the SSEA4-positive or SSEA4-negative subpopulation was determined by flow cytometry, which indicated a regulation of SSEA4 expression back to the initial level during the growth phase in vivo. (PNG 93 kb) Additional file 8: Table S2. Treatment history of breast cancer patients. Detailed description of treatment history of breast cancer patients. (XLSX 11 kb) Additional file 9: Table S3. Results of microarray analysis. Results of gene and miRNA expression profiling, Gene Ontology analysis, and miRNA target prediction. (XLSX 118 kb) Additional file 10: Figure S6. SSEA4-positive breast cancer cells show differential expression of genes pointing toward a mesenchymal state as well as increased expression of members of the SLC and multidrug resistance ATP-binding cassette transporter families, but not of stem cell associated transcripts. (**a**) In the SSEA4-positive cell fraction, genes characteristic of an epithelial state showed decreased expression compared with the SSEA4-negative fraction. In contrast, genes characteristic of a mesenchymal state showed increased expression compared with the SSEA4-negative fraction. (**b**) In the SSEA4-positive cell fraction, members of the SLC and multidrug resistance ATP-binding cassette transporter families showed increased expression compared with the SSEA4-negative fraction. (**c**) Stem cell markers were not consistently regulated among the SSEA4-positive and SSEA4-negative cell fractions. The housekeeping gene *GAPDH* showed no significant regulation among the subpopulations. Each bar represents the log_2_ expression ratio of the SSEA4-positive fraction relative to the SSEA4-negative fraction for the respective tumor model. (TIFF 655 kb) Additional file 11: Figure S7. Validation of miRNA candidates using a flow cytometry–based 39-plex miRNA assay. (**a**) Cluster analysis of expression ratios (log_2_-transformed) obtained from hybridization of SSEA4-positive (pos) and SSEA4-negative (neg) samples. The miRNAs that were significantly downregulated in SSEA4-positive cells based on the microarray analysis are highlighted with a *blue bar*. (**b**) Comparison of miRNA bead assay (BA) and microarray data (MA; average of all three samples). Seven miRNAs that were differentially expressed between SSEA4-positive and SSEA4-negative cells, as well as three miRNAs (miR-30b-5p, miR-29a-3p, and miR-16-5p) expressed at a similar level in both cell types, are shown. The bead assay results correlated well with the microarray data. (PNG 509 kb) Additional file 12: Figure S8. SSEA4-positive breast cancer cells show decreased expression of miRNAs inhibiting EMT inducers. Expression ratios of the 12 miRNAs targeting the key mesenchymal regulator and indicator genes *ZEB1*, *ZEB2*, *fibronectin 1*, *Snail1*, *Snail2*, and *Twist*. Each bar represents the log_2_ expression ratio of the SSEA4-positive fraction relative to the SSEA4-negative fraction for the respective tumor model. (TIFF 201 kb) Additional file 13: Figure S9. Expression of SSEA4 in RCC and healthy kidney tissue. Primary RCC and healthy kidney tissues from the same patient were dissociated and analyzed by multiparametric flow cytometry. Doublets were excluded by FSC-A/FSC-H gating (**a**); debris was excluded by FSC/SSC gating (**b**); dead cells were excluded by gating off PI^+^ events (**c**); and lineage-positive cells were excluded by gating on α-Lin-FITC–negative events (**d**). In each patient, healthy and tumor tissues were analyzed in parallel in one labeling reaction. Therefore, one of the samples was labeled using a UV dye, allowing for subsequent separation of the events of each sample by gating on the VioBlue channel fluorescence intensity (**e**–**h**). In all of the analyzed patients (*n* = 3), the expression of SSEA4 was strongly increased in the tumor tissue as compared with the respective healthy tissue, with almost all tumor cells expressing SSEA4 in two of the patients (**f**–**h**). *α-Lin-FITC = CD45-FITC, CD31-FITC, CD235a (glycophorin A)-FITC. (PNG 717 kb)

## Abbreviations

AC: doxorubicin/cyclophosphamide
APC: allophycocyanin
CT: chemotherapy
CTC: circulating tumor cell
DAPI: 4′,6-diamidino-2-phenylindole
EMT: epithelial–mesenchymal transition
EpCAM: epithelial cell adhesion molecule
ER: estrogen receptor
FIGO: International Federation of Gynecologists and Obstetricians
FITC: fluorescein isothiocyanate
FSC: forward scatter
FSC-A: forward scatter area
FSC-H: forward scatter height
GEO: Gene Expression Omnibus
GSL: glycosphingolipid
HBCx: human breast cancer xenograft
IC_50_: half-maximal inhibitory concentration
MFI: median fluorescence intensity
miRNA: microRNA, mIR
mRA: messenger RNA
PDX: patient-derived xenograft
PE: pleural effusion
PI: propidium iodide
PR: progesterone receptor
RCC: renal cell carcinoma
siRNA: small interfering RNA
SSC: side scatter
SSEA4: sialyl-glycolipid stage-specific embryonic antigen 4
ST3GAL2: CMP-*N*-acetylneuraminate-β-galactosamide-α-2,3-sialyltransferase 2
TGF: transforming growth factor
TM: treatment
TNBC: triple-negative breast cancer
