# Supplementary material for: The sialyl-glycolipid stage-specific embryonic antigen 4 marks a subpopulation of chemotherapy-resistant breast cancer cells with mesenchymal features
Source: Breast Cancer Res. 2015 Nov 25;17:146. doi: 10.1186/s13058-015-0652-6 (PMC4660783; doi:10.1186/s13058-015-0652-6)
Supplement: Additional file 1: — Supplemental experimental procedures. Detailed description of materials and methods. (DOCX 62 kb) [file 13058_2015_652_MOESM1_ESM.docx]

### Supplemental Experimental Procedures

*Primary tissue material and xenotransplantation*

Female athymic nude mice (Hsd:Athymic Nude-*Foxn1^nu^,* Harlan), 5-week-old, were maintained under specific pathogen-free conditions at the animal facility of the Center for Exploration and Experimental Functional Research (CERFE, Evry, France). Animal care and housing were in accordance with the guidelines provided by the regional ethics committee for animal experimentation (Ile de France, France). Human breast cancer xenografts were established from patient’s primary tumor surgical specimens, by grafting tumor fragments into the interscapular fat pad of athymic nude mice, and maintained through *in vivo* passages as previously described [9].

The tumor models used in this study were at passage 18 (HBCx-17), 21 (HBCx-10), 11 (HBCx-6), and 14 (HBCx-14). Tumors of the same passage were transplanted subcutaneously onto 5-10 mice (donor mice). When these tumors reached a volume of 1000 to 2000 mm^3^, donor mice were sacrificed by cervical dislocation. Tumors were excised aseptically and dissected by removing necrotic areas and cutting the tumor tissue into pieces of approximately 20 mm^3^. The tumor fragments were immediately transferred in EMEM culture medium (Life Technologies). Prior to grafting, athymic nude mice (receptor mice) at the age of 6-8 weeks were anaesthetized with ketamine/xylazine as described before [9]. The skin, previously made aseptic with chlorhexidine solution, was incised at the level of the interscapular region, and a 20 mm^3^ tumor fragment was placed in the subcutaneous tissue. The skin incision was closed with clips. The mice used for each experiment were implanted on the same day. The clips were removed two weeks after transplantation. At that time point, tumor growth observation was started by measuring tumor volume twice a week.

*Chemotherapeutic treatment*

Mice with tumors of a size between 150-350 mm^3^ were included in each experiment. Adriamycin (ADRIBLASTIN® RD, Pfizer) and cyclophosphamide (ENDOXAN®, Baxter) solutions were prepared in 0.9% NaCl. Both drugs were administered on the same day via intraperitoneal injection at a dose of 2 mg/kg (adriamycin) and 100 mg/kg (cyclophosphamide). To obtain a complete response for models HBCx-17 and HBCx-6, the same dose of Adriamycin/cyclophosphamide chemotherapy was applied a second time, three weeks after the first injection. Adriamycin/cyclophosphamide chemotherapy was applied to 68 mice of tumorgraft model HBCx-17, 32 mice of HBCx-10, 35 mice of HBCx-6 and 30 mice of HBCx-14 model not including the control group.

Tumor volume and mouse weight were monitored twice a week. Tumor size measurements were done with a calliper by measuring the longest and the shortest diameter of the tumor. To calculate tumor volume, the formula Tumor volume (mm^3^) = [length (mm) x ((width (mm))^2^]/2 was used, where the length and the width are the longest and the shortest diameters of the tumor, respectively. The mice were excluded from the study and sacrificed, if any of these criteria were present: 1) body weight loss ≥ 20% compared to the first day of treatment for 72 consecutive hours 2) general alteration of behaviour or clinical signs 3) tumor volume ≥ 2000 mm^3^.

At least six pre-treated tumors for each model (untreated tumors of size between 150 mm^3^ and 350 mm^3^) were harvested on the day of the first chemotherapy treatment. Residual tumors were considered the chemotherapy-treated tumors that regressed to a volume between 14 and 63 mm^3^. When the majority of the treated tumors attained that size, about 2/3 of them were harvested and the remaining tumors were left to re-grow. Regrown tumors were harvested when their average size recovered to the initial pre-treated one (150 mm^3^ - 350 mm^3^). Untreated tumors bigger than 350 mm^3^ were collected for analyses the same day of the treated tumors to check whether tumor size can also influence marker expression.

Whole tumors were dissected out together with the surrounding stromal tissue and were placed in a 50 mL falcon tube containing MACS^®^ tissue storage solution (Miltenyi Biotec). Samples were transferred into a refrigerated box and were sent by an overnight shipment to Miltenyi Biotec (Bergisch Gladbach, Germany) for analysis.

*Depletion of mouse cells*

Xenograft tumors contain a substantial amount of murine origin cells, like white and red blood cells, fibroblasts, and endothelial cells. These cell can bias downstream analysis like cell culture experiments and mRNA or miRNA expression profiling since the overall amount as well as composition of these contaminating cells is highly variable among tumor specimens. To avoid any bias caused by this, all mouse cells were depleted using the Mouse Cell Depletion Kit (Miltenyi Biotec) according to the manufacturer’s instructions.

*Isolation of SSEA4+ and SSEA4- tumor cell subpopulations*

SSEA4^+^ and SSEA4^-^ tumor cell subpopulations were isolated by magnetic activated cell sorting (MACS^®^). After dissociation and depletion of mouse cells, the cells were resuspended in PEB buffer (PBS, pH 7.2, 0.5% bovine serum albumin, and 2 mM EDTA; prepared by diluting MACS BSA Stock Solution 1:20 with autoMACS^®^ Rinsing Solution) at a concentration of 1x10^7^ cells per 100 µl. After adding 10 µl SSEA4-PE (Miltenyi Biotec) per 100 µl, the suspension was incubated at 4°C under continuous agitation (MACSmix Tube Rotator, Miltenyi Biotec) for 10 min. Then the cells were pelleted at 300 g for 10 min, resuspended in 80 µl PEB per 1x10^7^ cells, 20 µl of anti-PE MicroBeads (Miltenyi Biotec) were added and incubated at 4°C under continuous agitation for 15 min. Cells were washed in 1 ml PEB per 1x10^7^ cells, pelleted and resuspended in 500 µl PEB per separation. SSEA4^+^ tumor cells were isolated using an MS-column (Miltenyi Biotec), SSEA4^-^ tumor cells were isolated using an LD-column (Miltenyi Biotec). Purity of the isolated cells was evaluated using flow cytometry. For microarray analysis, cells were pelleted and lysed in Qiazol® (QIAGEN).

*Microarray hybridization*

RNA quality and integrity were determined using the Agilent RNA 6000 Nano Kit on the Agilent 2100 Bioanalyzer (Agilent Technologies). RNA was quantified by measuring A260nm on the ND-1000 Spectrophotometer (NanoDrop Technologies).

For mRNA expression profiling, sample labeling was performed as detailed in the Agilent “One-Color Microarray-Based Gene Expression Analysis protocol (version 6.6, part number G4140-90040). Briefly, 100 ng of each total RNA samples was used for the amplification and labeling step using the Agilent Low Input Quick Amp Labeling Kit (Agilent Technologies). Each amplification was processed as a technical duplicate. Yields of cRNA and the dye-incorporation rate were measured with the ND-1000 Spectrophotometer (NanoDrop Technologies). The hybridization procedure was performed according to the Agilent “One-Color Microarray-Based Gene Expression Analysis protocol (version 6.6, part number G4140-90040) using the Agilent Gene Expression Hybridization Kit (Agilent Technologies). Briefly, 0.6 µg Cy3-labeled fragmented cRNA in hybridization buffer was hybridized overnight (17 hours, 65 °C) to Agilent Whole Human Genome Oligo Microarrays 8x60K v2 (Design ID 039494) using Agilent’s recommended hybridization chamber and oven. Following hybridization, the microarrays were washed with the Agilent Gene Expression Wash Buffer 1 for 1 min at room temperature followed by a second wash with preheated Agilent Gene Expression Wash Buffer 2 (37 °C) for 1 min.

For miRNA expression profiling, sample labeling and hybridization was performed according to the “miRNA Complete Labeling and Hyb Kit” protocol (version 2.4, part number G4170-90011) using the miRNA Complete Labeling and Hyb Kit (Agilent Technologies). Briefly, Cy3-labeled RNA in hybridization buffer was hybridized overnight (20 hours, 55 °C) to Agilent Human microRNA Microarrays 8x60K v19 (Design ID 046064) using Agilent’s recommended hybridization chamber and oven. Following hybridization, the microarrays were washed with the Agilent Gene Expression Wash Buffer 1 for 5 min at room temperature followed by a second wash with preheated Agilent Gene Expression Wash Buffer 2 (37 °C) for 5 min.

Fluorescence signals of the hybridized Agilent Microarrays were detected using Agilent’s Microarray Scanner System G2505C (Agilent Technologies). The Agilent Feature Extraction Software (FES) 10.7.3.1 was used to read out and process the microarray image files.

*Microarray data analysis*

The mRNA data discussed in this publication have been deposited in NCBI's Gene Expression Omnibus [12] and are accessible through GEO Series accession number GSE57705 (http://www.ncbi.nlm.nih.gov/geo/query/acc.cgi?acc= GSE57705).

The Agilent Feature Extraction Software (FES) version 10.7.3.1 was used to read out and process the microarray image files. After probe summarization, quantile normalization and log2 transformation, differentially expressed genes were identified by a combination of effect size and statistical significance.

For the analysis of the individual tumor models, only reporters with an at least 1.5-fold average up- or downregulation in SSEA4+ samples compared to control samples and an unadjusted p-value (Student’s t-test, two-tailed, equal variance) ≤0.05 were considered relevant. In an additional filtering step, only Agilent reporters with signal intensity values significantly above local background (p<0.01 as calculated by the Rosetta Resolver® gene expression data analysis system) in both samples of the group with higher average expression were selected [1].

For the identification of genes consistently regulated among all tumor models, paired t-tests were conducted based on the average signal intensities calculated from the technical replicates of each cell fraction. Further selection steps include filtering for effect size and detectability as described above. Using discriminatory gene analysis, 736, 985, and 1384 genes were identified as being significantly upregulated (p < 0.01, ≥1.5-fold) and 127, 567, and 334 genes were significantly downregulated (p < 0.01, ≤-1.5-fold) in tumor models HBCx6, HBCx10, and HBCx14, respectively .

The lists of up- and downregulated genes were separately subjected to a functional annotation analysis. For this purpose, Gene Ontology terms were summarized to superordinate categories and assigned to the differentially expressed genes [18]. Term enrichment relative to the expected background distribution was scored using Fisher’s exact test with Benjamini–Hochberg correction (p < 0.05).

The miRNA data discussed in this publication have been deposited in NCBI's Gene Expression Omnibus [12] and are accessible through GEO Series accession number GSE57705 (http://www.ncbi.nlm.nih.gov/geo/query/acc.cgi?acc= GSE57705).

The intensity data of the individual microarrays were subjected to probe summarization, thresholding (0.1), log2-transformation, and quantile normalization (GeneSpring v12.6, Agilent Technologies). Based on the average signal intensities calculated from the technical replicate samples for each cell fraction, median fold-change values for the comparison of SSEA4-positive and negative cell fractions were determined and a paired t-test (two-tailed, equal variance) was conducted. Only miRNAs with an at least two-fold average up- or downregulation in SSEA4+ samples compared to controls and an unadjusted t-test p-value ≤0.05 were considered relevant. In an additional filtering step, only miRNAs with signal intensity values ≥10 light units in at least four out of six replicate samples of the group with higher average expression were selected.

Hierarchical clustering of median-centered log2 intensity values was conducted using Euclidean distance metric (complete linkage) [2].

For the analysis of miRNA-mRNA interactions, miRNA-mRNA target pairs were downloaded for six different computational target prediction tools (miRDB, <http://mirdb.org/miRDB/>; PicTar, <http://pictar.mdc-berlin.de/>; Elmmo, <http://www.mirz.unibas.ch/ElMMo3/>; miRanda, <http://www.microrna.org/microrna/home.do>; PITA, <http://genie.weizmann.ac.il/pubs/mir07/>; and TargetScan, <http://www.targetscan.org/>). MicroRNA-mRNA target pairs were considered relevant only when predicted by at least two of these tools.

*Analysis of miRNAs expression using a flow cytometry-based 39-plex miRNA assay*

The MACSPlex miRNA Cancer Kit (Miltenyi Biotec) was used to validate miRNAs detected as differentially expressed based on microarray results. The assay was carried out according to the manufacturer’s protocol. Briefly, 500 ng miRNA per sample were fluorescently labelled and hybridized to color-coded beads carrying miRNA specific probes. Fluorescence signals of the hybridized beads were detected using the MACSQuant Analyzer (Miltenyi Biotec). Data were normalized using a spike-in control.

*Clinical data analysis*

To evaluate the prognostic value of candidate genes, the publically available Kaplan-Meier Plotter (http://kmplot.com/analysis/) was used. Briefly, gene expression data, distant metastasis free survival and relapse free and overall survival information are downloaded from GEO (Affymetrix microarrays only), EGA and TCGA. The background database is handled by a PostgreSQL server, which integrates gene expression and clinical data simultaneously and is manually curated. To analyze the prognostic value of a particular gene, the patient samples are split into two groups according to the median of the proposed biomarker. The two patient cohorts are compared by a Kaplan-Meier survival plot, and the hazard ratio with 95% confidence intervals and logrank P value are calculated [13,14].

*In vitro cytotoxicity assays*

SSEA4-positive, SSEA4-negative, and unsorted tumor cells were seeded in 96-well flat-bottom plates (1250 to 5000 cells/well) in Advanced DMEM medium (Life Technologies) supplemented with 8% fetal bovine serum (Lonza), 2 mM GlutaMax (Lonza), and 100 U/µl penicillin-streptomycin (Life Technologies). The cells were incubated for two days at 37°C and 5% CO_2_, after which (day 0) the cells were treated with seven different standard chemotherapy drugs: adriamycin (Sigma), mafosfamide (Niomech), 5-FU (Sigma), cisplatin (Sigma), etoposide (Sigma), topotecan (Sigma), and irinotecan (Sigma).

To obtain a dose response curve, each drug was tested at serial concentrations ranging from 10 µM to 0.00051 µM (adriamycin), 100 µM to 0.0051 µM (mafosfamide), 500 µM to 0.025 µM (5-FU), 200 µM to 0.01 µM (cisplatin), 100 µM to 0.005 µM (etoposide), 20 µM to 0.001 µM (topotecan), and 100 µM to 0.005 µM (irinotecan). Cell viability was analyzed 72 hours after addition of drugs using CellTiter-Glo® Luminescent Cell Viability Assay Kit (Promega) according to the manufacturer’s instructions. Luciferase activity was measured on a luminometer (PerkinElmer^®^ EnVision^TM^).

The assay was performed in duplicates or triplicates for each tumor cell fraction. The cell viability and number of untreated cells was analyzed at day 0 and 3, thus allowing to determine the doubling time of each cell fraction. Cell viability was calculated as a percentage of the mean of the ATP values of the replicate compared to the mean ATP values of all not-treated cell wells, which was defined as the 100% of viability. When possible, an IC50 value was determined as the concentration of drug that reduces cell viability by 50%. IC50 values were calculated using Microsoft Excel.

*siRNA mediated knockdown*

A mixture of four small interfering RNAs (ON-TARGET plus SMART pool siRNAs) for *ST3GAL2*, *ST3GAL3*, and CD133 were obtained from Thermo Scientific (Pittsburgh, PA). The siRNA pool specific for CD133 was used as a positive control. The siRNA sequences were:

*ST3GAL2*

5′-AUGUGUGUGAUGAGGUGAA-3´

5′-CAGCCUUCUUCAAGUAUAU-3´

5′-ACAGCCACUUUGACGGUAA-3´

5′-AGAUAGUGCCUGGCGAGAA-3´

*ST3GAL3*

5′-GGUGAAAGCUCGCGUCAUC-3´

5′-UGGAUGACUCCUUUCGCAA-3´

5′-UCUUGGUAUUUGUGCGCAA-3´

5′-CGCACAAUAUCCAGCGAGA-3´

CD133

5′-UCACAAUCCUGUUAUGACA-3´

5′-GAAGUAUGGGAGAACAAUA-3´

5´-GAACAAGUUUACAGUGACU-3´

5´-GCUAAGUACUAUCGUCGAA-3´

Non-targeting siRNA #1 was used as negative control. 1x10^5^ cells were seeded in each well of a 24 multi-well plate. After 24 h and 70% confluence, the siRNA molecules (final concentration 25 nM) were transfected using 1.5 μL of Lipofectamine RNAiMAX (Invitrogen, Carlsbad, CA) per well according to manufacturer's instructions. Forty-eight and 72 hours after transfection with siRNA against control scramble, CD133, *ST3GAL2*, *ST3GAL3*, and *ST3GAL2* plus *ST3GAL3*, the knockdown efficiency was examined by western blot and by flow cytometric analysis.

*SSEA4 Immunohistochemistry*

Snap-frozen tissues were sliced in 10 μm thick sections and stored at -80°C for at least 24 hours. Sections were thawed at RT for 1.5 h and fixed for 30 min using 4% PFA. After washing in PBS, sections were incubated in 10% goat serum for 2 h to block nonspecific binding. After two additional washes in PBS, the sections were incubated with humanized monoclonal antibody specific for SSEA4 diluted at 20 μg/mL in blocking buffer at 4°C overnight. After three washes for 10 min in PBS, the sections were incubated with an Alexa Fluor 594 Goat Anti-Human H+L (Life Technologies) coupled secondary antibody diluted at 1:300 in blocking buffer for 1.5 h followed by washing in PBS/0.2% triton X100. Sections were counterstained with TOTO-3 (Life Technologies) diluted 1:800 in PBS.

*SSEA-4 Cross-Blocking analysis*

Cross-blocking experiments were performed to assess the binding specificity of the Anti-SSEA-4 antibody derived from clone REA101 (Miltenyi Biotec, 130-100-635) in comparison to one derived from clone MC-813-70 (Stemgent, 09-0003). Single cell suspensions of human iPSC were generated using TrypLE^TM^ (Thermo Fisher) and resupended in PEB. 0.5 million cells were either directly fluorescently labelled using antibody-PE conjugates from clone REA101 (25ng/ml) or MC-813-70 (333ng/ml) for 10 min at 4°C, or after cells had been blocked by pre-incubation with 100µg/ml unconjugated antibody of the alternative clone for 10 min at 4°C. In the latter case, staining antibodies were added to the blocked cell suspension without an intermediate washing step. After immunofluorescent labeling, cells were washed once, resuspended in PEB and analyzed by flow cytometry using a MACSQuant**^®^** Analyzer (Miltenyi Biotec).

*qPCR*

Total RNA was extracted using the miRNeasy mini kit (Qiagen), followed by reverse transcription of RNA with the high capacity cDNA reverse transcription kit (Applied Biosystems, Life Technologies) according to the manufacturer’s instructions. Quantitative Real-time PCR was performed using 10 ng of starting RNA and TaqMan Universal PCR Master Mix (Applied Biosystems, Life Technologies). FAM-MGB dye-conjugated TaqMan probes (Applied Biosystems, Life Technologies) for SNAI1 (Hs00195591_m1), FN1 (Hs00365052_m1), ZEB2 (Hs00207691_m1), CLDN3 (Hs00265816_s1), GAPDH (HS02758991_g1) and PPIA (Hs04194521_s1) were used to obtain expression data with the Viia7 Real-Time PCR System (Applied Biosystems, Life Technologies). The ViiA7 software 1.1 was used for data acquisition and analysis. Relative quantitation of gene expression was calculated on triplicates using the comparative CT (ΔΔCT) method. Data analysis was performed using GraphPad Prism software (V6, GraphPad Software Inc.).

*Cultivation of human induced pluripotent stem cells (iPSC)*

Human foreskin fibroblasts-derived iPSC were cultivated on Matrigel (BD Bioscience) in StemMACS^TM^ iPS-Brew XF media (Miltenyi Biotec). Cells were passaged with TrypLE^TM^ (Thermo Fisher) every 4-5 days. Single cells were replated at a density of 8,000-12,500 cells/cm^2^. Media was supplemented with ROCK-inhibitor (Thiazovivin 2 µM, Miltenyi Biotec) for the first 48h. Afterwards, media was changed daily.

### Supplemental References

1. Weng L, Dai H, Zhan Y, He Y, Stepaniants SB, Bassett DE: **Rosetta error model for gene expression analysis**. *Bioinformatics (Oxford, England)* 2006, **22**(9):1111-1121.

2. Saeed AI, Sharov V, White J, Li J, Liang W, Bhagabati N, Braisted J, Klapa M, Currier T, Thiagarajan M *et al*: **TM4: a free, open-source system for microarray data management and analysis**. *BioTechniques* 2003, **34**(2):374-378.
