# Supplementary material for: The sialyl-glycolipid stage-specific embryonic antigen 4 marks a subpopulation of chemotherapy-resistant breast cancer cells with mesenchymal features
Source: Breast Cancer Res. 2015 Nov 25;17:146. doi: 10.1186/s13058-015-0652-6 (PMC4660783; doi:10.1186/s13058-015-0652-6)
Supplement: Additional file 2: Table S1. — Antibodies used for the screening approach. Detailed description of used antibodies. (DOCX 46 kb) [file 13058_2015_652_MOESM2_ESM.docx]

**Table S1: Antibodies used for the screening approach**

|  |  |  |  |
| --- | --- | --- | --- |
|  |  |  |  |
| **Antigen** | **Clone** | **Vendor** | **Titer** |
|  |  |  |  |
|  |  |  |  |
| AN2/MCSP | 1E6.4 | Miltenyi Biotec | **1:11** |
| ABCB5 | Polyclonal rabbit IgG | Bioss | **1:50** |
| CaSR | Polyclonal rabbit IgG | Enzo Life Science | **1:50** |
| CD9 | M-L13 | BD Biosciences | **1:11** |
| CD10 | 97C5 | Miltenyi Biotec | **1:11** |
| CD15/SSEA1 | VIMC6 | Miltenyi Biotec | **1:11** |
| CD20 | LT20 | Miltenyi Biotec | **1:11** |
| CD24 | 32D12 | Miltenyi Biotec | **1:11** |
| CD26 | FR 10-11G9 | Miltenyi Biotec | **1:11** |
| CD34 | AC136 | Miltenyi Biotec | **1:11** |
| CD38 | IB6 | Miltenyi Biotec | **1:11** |
| CD44 | DB105 | Miltenyi Biotec | **1:11** |
| CD49a | TS/27 | BioLegend | **1:20** |
| CD49b | Y418 | eBioscience | **1:20** |
| CD49c | ASC-1 | BioLegend | **1:20** |
| CD49d | MZ18-24A9 | Miltenyi Biotec | **1:11** |
| CD49e | NKI-SAM1 | Miltenyi Biotec | **1:11** |
| CD49f | GoH3 | Miltenyi Biotec | **1:11** |
| CD61 | Y2/51 | Miltenyi Biotec | **1:11** |
| CD66 (a,c,d,e) | TET2 | Miltenyi Biotec | **1:11** |
| CD71 | AC102 | Miltenyi Biotec | **1:11** |
| CD90 | DG3 | Miltenyi Biotec | **1:11** |
| CD105 (Endoglin) | 43A4E1 | Miltenyi Biotec | **1:11** |
| CD117 | AC126 | Miltenyi Biotec | **1:11** |
| CD122 | Tu27 | BioLegend | **1:11** |
| CD133/1 | W6B3C1 | Miltenyi Biotec | **1:11** |
| CD133/2 | 293C3 | Miltenyi Biotec | **1:11** |
| CD138 | B-B4 | Miltenyi Biotec | **1:11** |
| CD146 | 541-10B2 | Miltenyi Biotec | **1:11** |
| CD166 | 3A6 | BioLegend | **1:6** |
| CD271 (NGF Receptor) | ME20.4-1.H4 | Miltenyi Biotec | **1:11** |
| CD309 (KDR/VEGF-R2) | ES8-20E6 | Miltenyi Biotec | **1:11** |
| CD324 (Ecad) | 67A4 | Miltenyi Biotec | **1:11** |
| CD325 (Ncad) | 8C11 | eBioscience | **1:11** |
| CD326 (EpCAM) | HEA-125 | Miltenyi Biotec | **1:11** |
| CD338 (ABCG2) | 5D3 | BD Biosciences | **1:20** |
| CD340 (Her2/neu) | 24D2 | BioLegend | **1:20** |
| DRD5 | Polyclonal rabbit IgG | Bioss | **1:100** |
| Lgr5 DA03 | DA03 | Miltenyi Biotec | **1:11** |
| ROR1 | 2A2 | Miltenyi Biotec | **1:11** |
| Sca1 | D7 | Miltenyi Biotec | **1:11** |
| SSEA4 | REA101 | Miltenyi Biotec | **1:11** |
| TGFbetaR | Polyclonal goat IgG | BD Biosciences | **1:11** |
| TRA-1-60 | REA157 | Miltenyi Biotec | **1:11** |
| TRA-1-81 | REA246 | Miltenyi Biotec | **1:11** |
|  |  |  |  |
|  |  |  |  |
