# Supplementary figures and images for: The sialyl-glycolipid stage-specific embryonic antigen 4 marks a subpopulation of chemotherapy-resistant breast cancer cells with mesenchymal features
Source: Breast Cancer Res. 2015 Nov 25;17:146. doi: 10.1186/s13058-015-0652-6 (PMC4660783; doi:10.1186/s13058-015-0652-6)

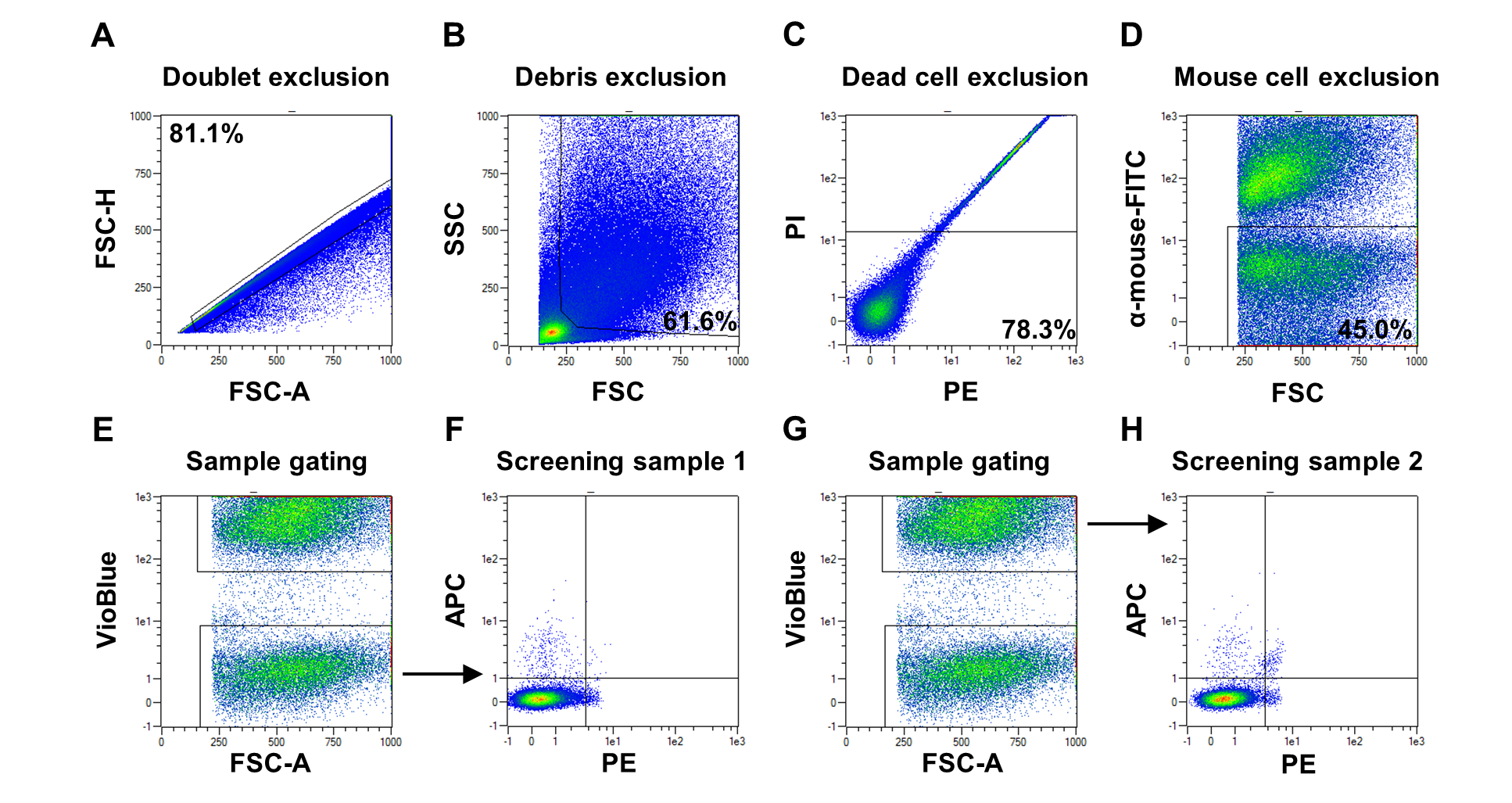

Supplement: Additional file 3: Figure S1. — Representative gating strategy for flow cytometry–based marker analysis of dissociated xenograft tumor tissue. Tumor tissue was dissociated to obtain a single-cell suspension while preserving cell surface epitopes. The sample was stained for mouse-specific markers to exclude cells of murine origin from the analysis as well as for the screening candidates and analyzed by multiparametric flow cytometry. Doublets were excluded by forward scatter (FSC) area/FSC height gating (a); debris was excluded by FSC/side scatter gating (b); dead cells were excluded by gating off propidium iodide–positive events (c); and mouse cells were excluded by gating on α-mouse-fluorescein isothiocyanate–negative events (d). When we screened two samples in parallel, we found that one of the samples was labeled using an ultraviolet dye, allowing for subsequent separation of the events of each sample by gating on the VioBlue channel fluorescence intensity (e–h). (PNG 736 kb) [file 13058_2015_652_MOESM3_ESM.png]

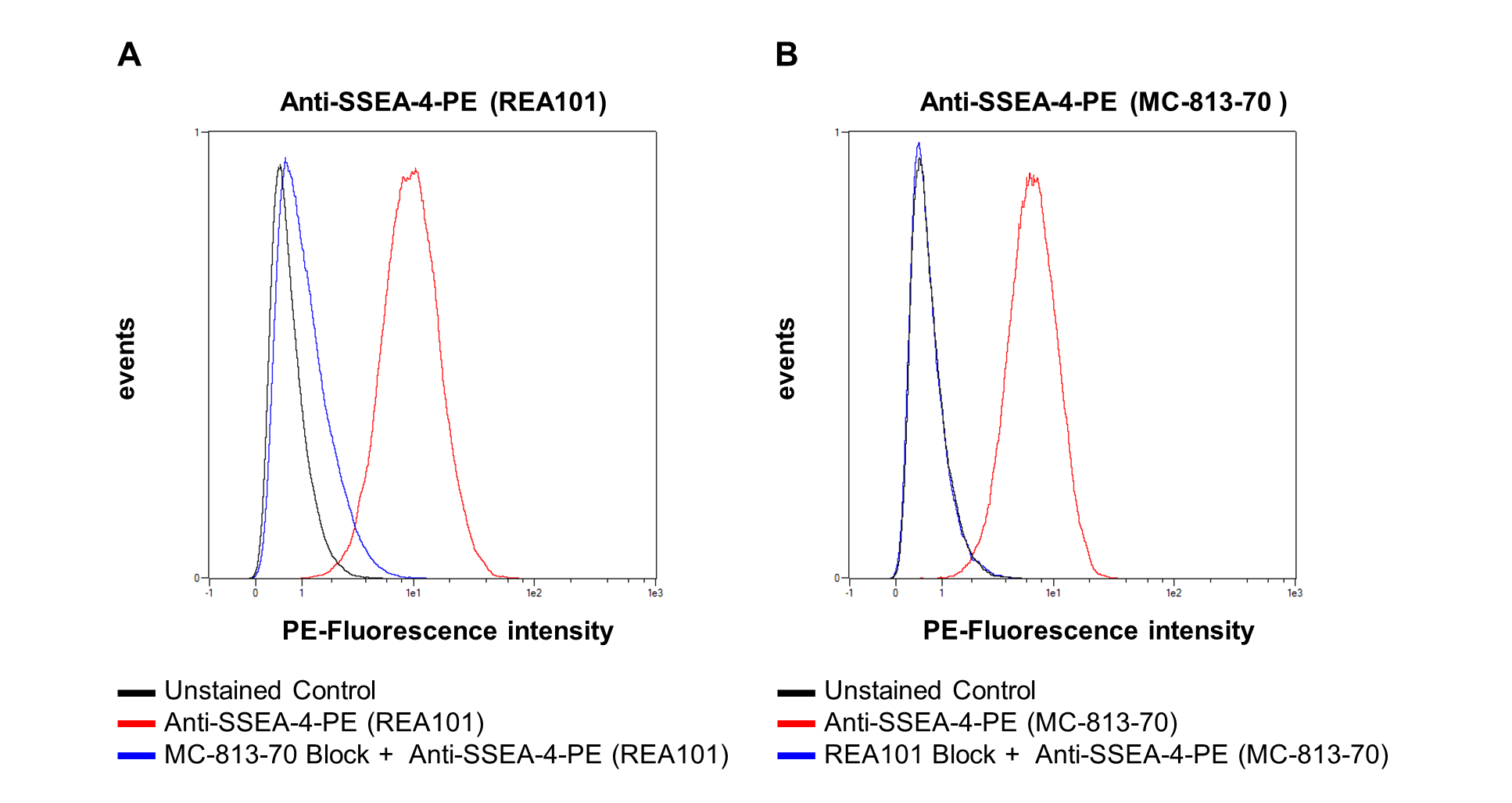

Supplement: Additional file 4: Figure S2. — Anti-SSEA4 antibodies derived from clone REA101 and MC-813-70 recognize the same epitope. Flow cytometric analysis of an antibody cross-blocking experiment on human induced pluripotent stem cells. Cells were either directly fluorescently labeled using anti-SSEA-4-phycoerythrin conjugates from clone REA101 (a) or MC-813-70 (b) or after cells had been blocked by preincubation with 100 μg/ml unconjugated antibody of the alternative clone (a, b). The fluorescent labeling of the REA101-derived anti-SSEA-4-phycoerythrin antibody was strongly diminished by blocking with an excess of MC-813-70 unconjugated antibody (a), while unconjugated REA101 caused a complete block of the fluorescent labeling of MC-813-70-derived anti-SSEA-4-phycoerythrin antibody (b). These results indicate that both antibodies recognize the same epitope and that the REA101-derived antibody has a higher functional affinity than the one derived from clone MC-813-70. (PNG 107 kb) [file 13058_2015_652_MOESM4_ESM.png]

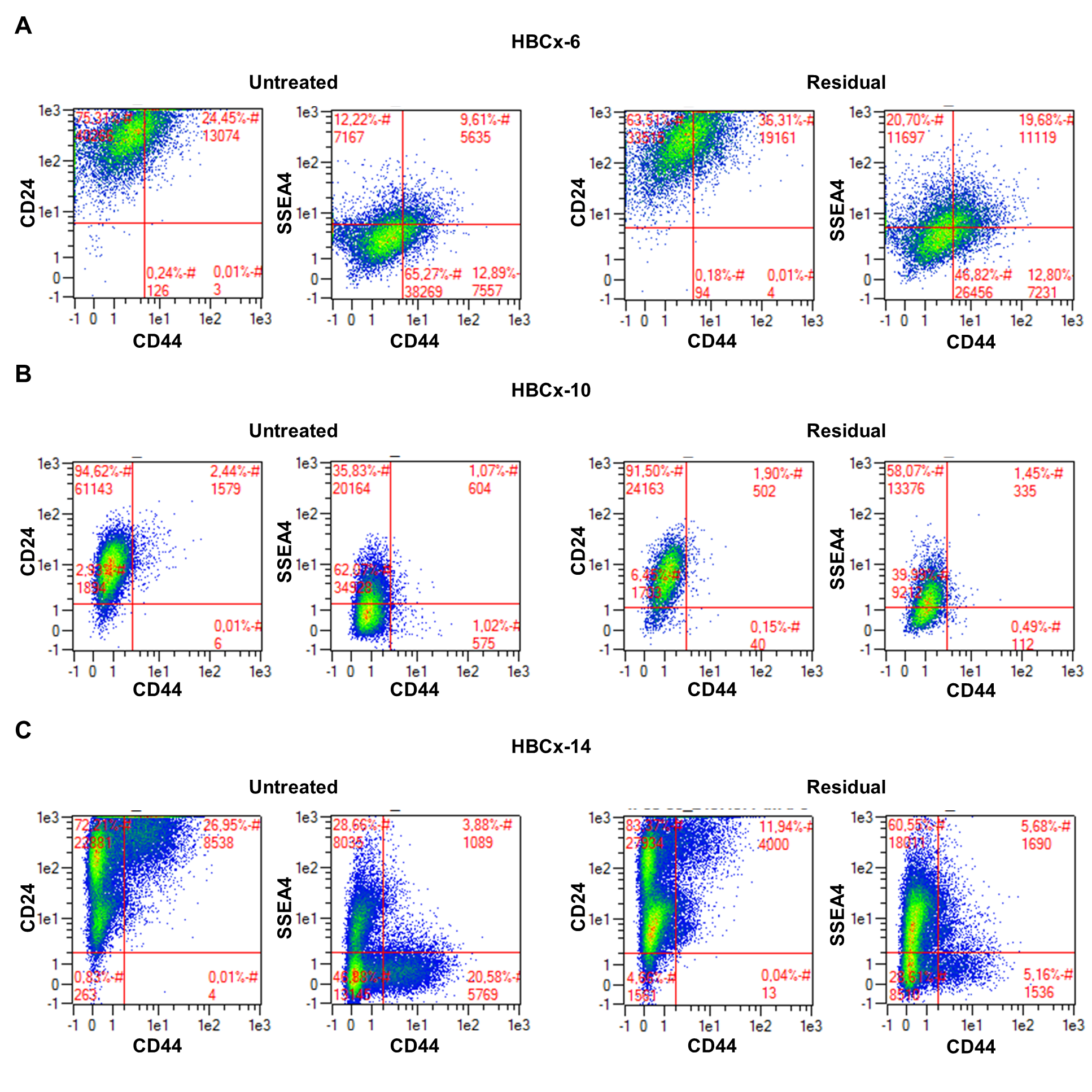

Supplement: Additional file 5: Figure S3. — Correlation among CD24-, CD44-, and SSEA4-expressing subpopulations. To address the correlation between CD24, CD44, and SSEA4 expression, we performed costaining of these markers on residual tumor nodules after AC chemotherapy and untreated tumors of three independent models: HBCx-6 (a), HBCx-10 (b), and HBCx-14 (c). Regulation of the three markers did not correlate among the treatment cycles. (PNG 1664 kb) [file 13058_2015_652_MOESM5_ESM.png]

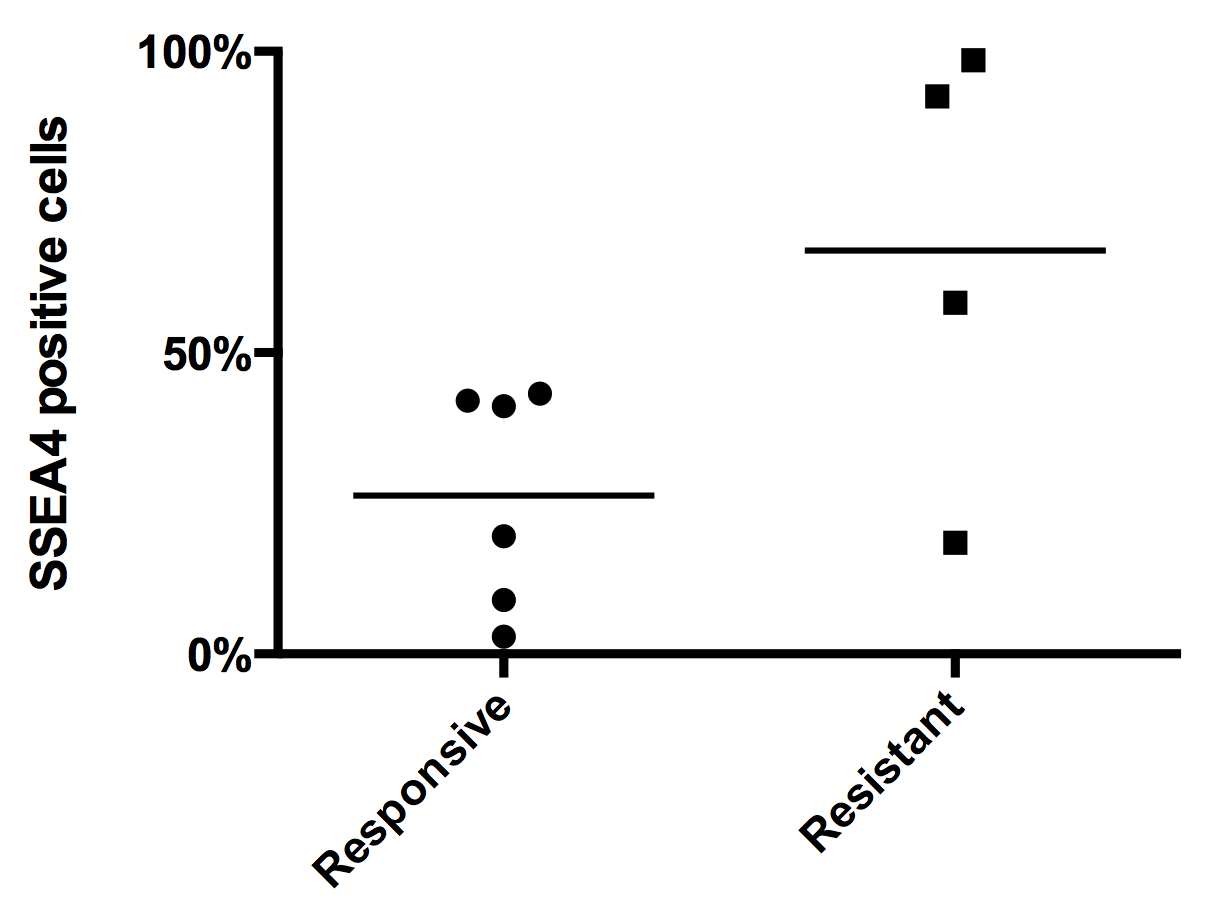

Supplement: Additional file 6: Figure S4. — Expression of SSEA4 in tumors responsive or resistant to chemotherapeutic treatment. Tumors responsive (n = 6) or resistant (n = 4) to AC treatment were analyzed for expression of SSEA4. Three of the four resistant tumor models showed higher percentages of SSEA4-positive cells than all of the six responsive tumors. In two of the resistant tumor models, almost all of the cells expressed SSEA4. (TIFF 61 kb) [file 13058_2015_652_MOESM6_ESM.tiff]

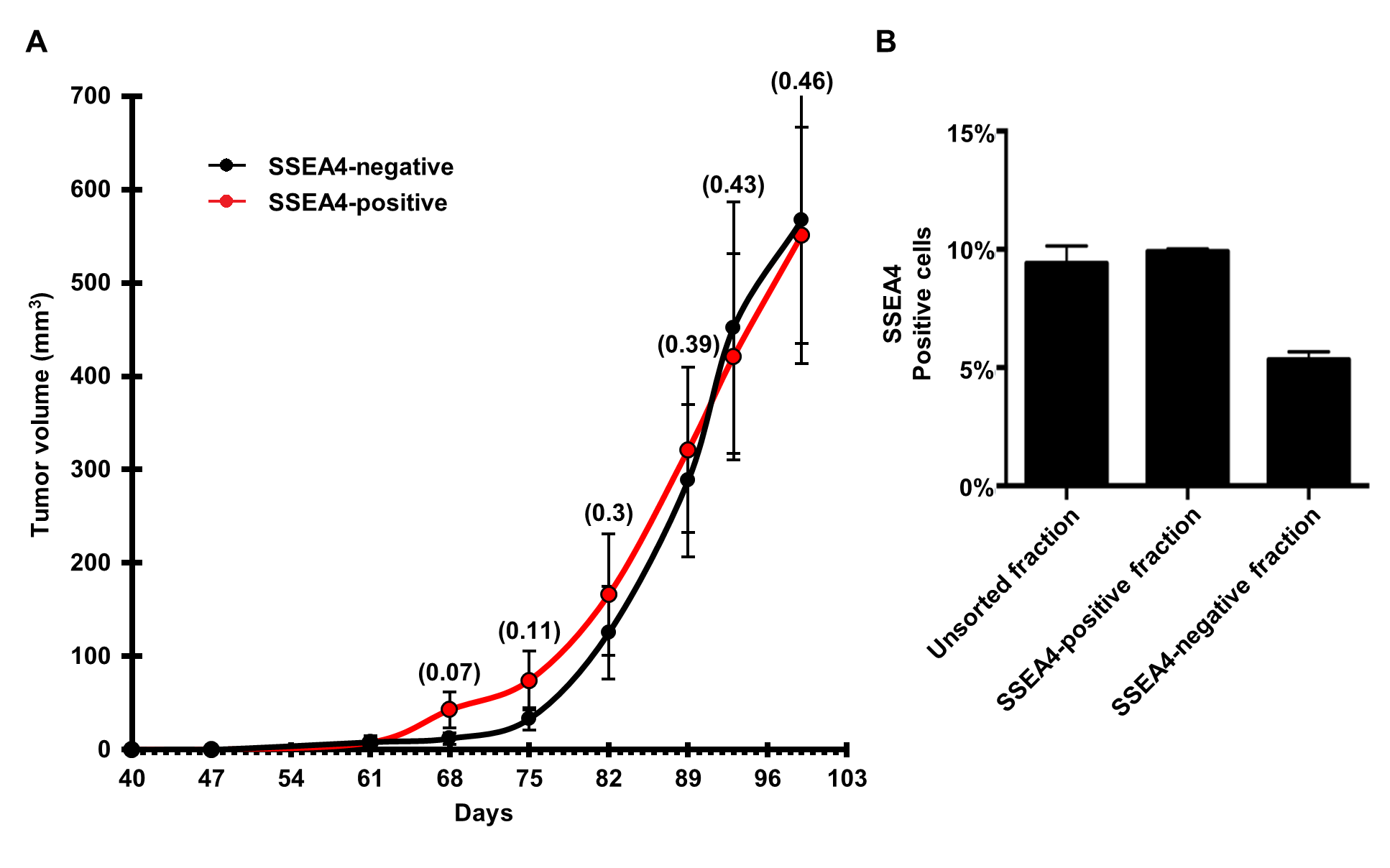

Supplement: Additional file 7: Figure S5. — Tumor-initiating capacity of the SSEA4-positive and SSEA4-negative subpopulation. (a) One hundred thousand freshly dissociated SSEA4-positive or SSEA4-negative cells were injected in two groups of eight mice each. Tumor volume was measured once per week, and the mean volume of both groups was calculated. The significance level (p value by t test) is indicated above each time point. (b) The frequency of SSEA4-expressing cells in the parental tumor model HBCx-14 and in tumors that originated from the SSEA4-positive or SSEA4-negative subpopulation was determined by flow cytometry, which indicated a regulation of SSEA4 expression back to the initial level during the growth phase in vivo. (PNG 93 kb) [file 13058_2015_652_MOESM7_ESM.png]

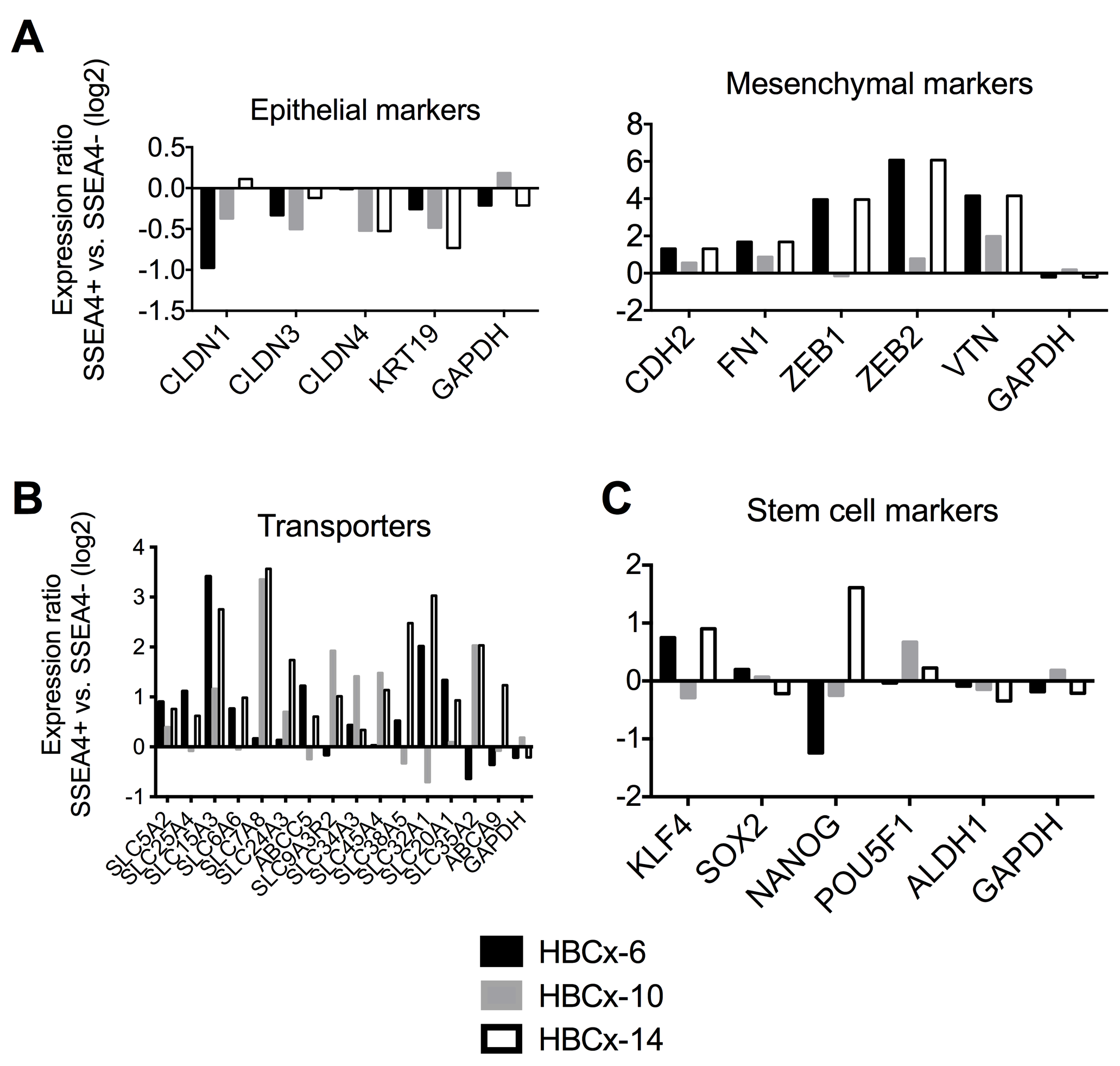

Supplement: Additional file 10: Figure S6. — SSEA4-positive breast cancer cells show differential expression of genes pointing toward a mesenchymal state as well as increased expression of members of the SLC and multidrug resistance ATP-binding cassette transporter families, but not of stem cell associated transcripts. (a) In the SSEA4-positive cell fraction, genes characteristic of an epithelial state showed decreased expression compared with the SSEA4-negative fraction. In contrast, genes characteristic of a mesenchymal state showed increased expression compared with the SSEA4-negative fraction. (b) In the SSEA4-positive cell fraction, members of the SLC and multidrug resistance ATP-binding cassette transporter families showed increased expression compared with the SSEA4-negative fraction. (c) Stem cell markers were not consistently regulated among the SSEA4-positive and SSEA4-negative cell fractions. The housekeeping gene GAPDH showed no significant regulation among the subpopulations. Each bar represents the log2 expression ratio of the SSEA4-positive fraction relative to the SSEA4-negative fraction for the respective tumor model. (TIFF 655 kb) [file 13058_2015_652_MOESM10_ESM.tiff]

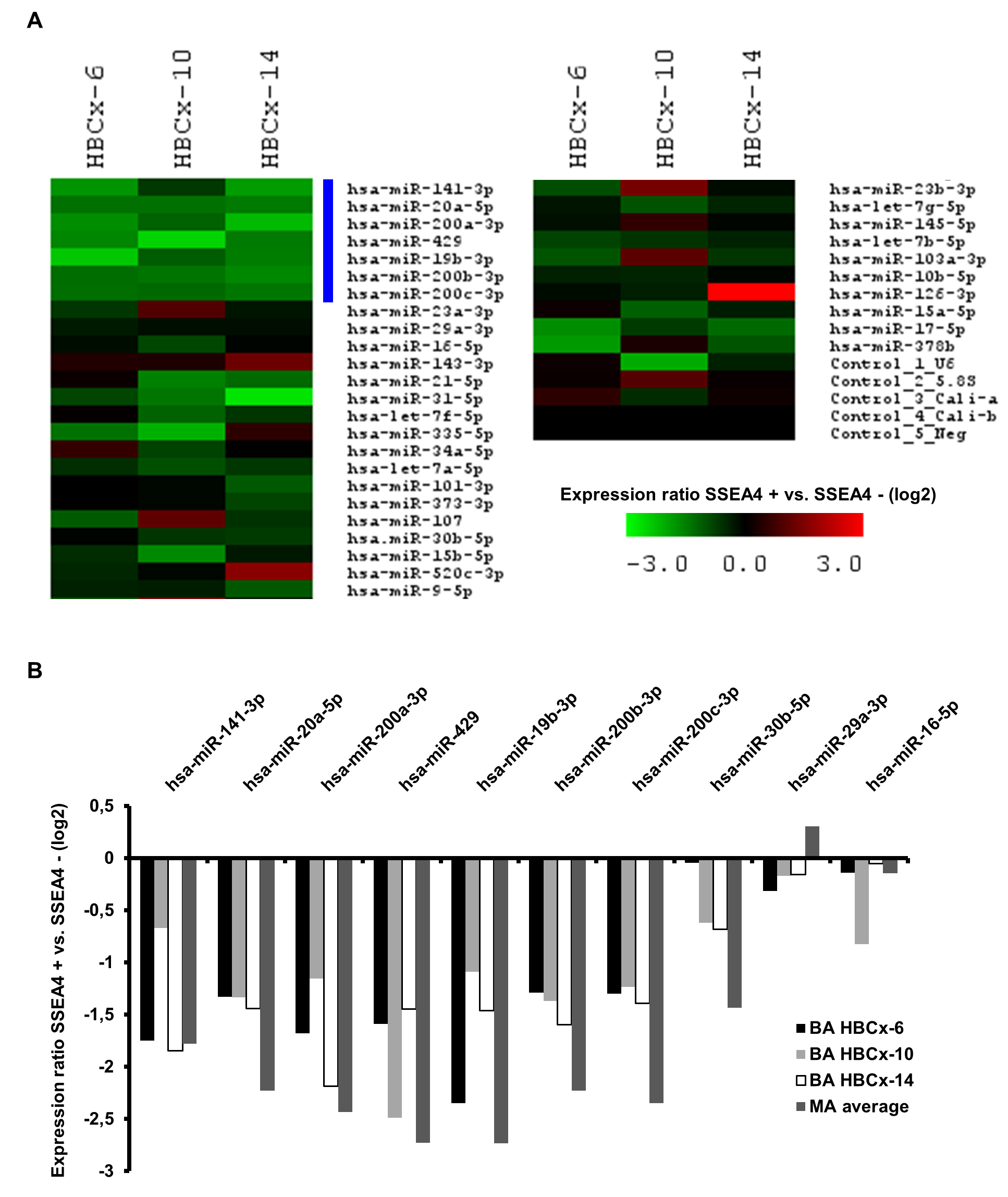

Supplement: Additional file 11: Figure S7. — Validation of miRNA candidates using a flow cytometry–based 39-plex miRNA assay. (a) Cluster analysis of expression ratios (log2-transformed) obtained from hybridization of SSEA4-positive (pos) and SSEA4-negative (neg) samples. The miRNAs that were significantly downregulated in SSEA4-positive cells based on the microarray analysis are highlighted with a blue bar. (b) Comparison of miRNA bead assay (BA) and microarray data (MA; average of all three samples). Seven miRNAs that were differentially expressed between SSEA4-positive and SSEA4-negative cells, as well as three miRNAs (miR-30b-5p, miR-29a-3p, and miR-16-5p) expressed at a similar level in both cell types, are shown. The bead assay results correlated well with the microarray data. (PNG 509 kb) [file 13058_2015_652_MOESM11_ESM.png]

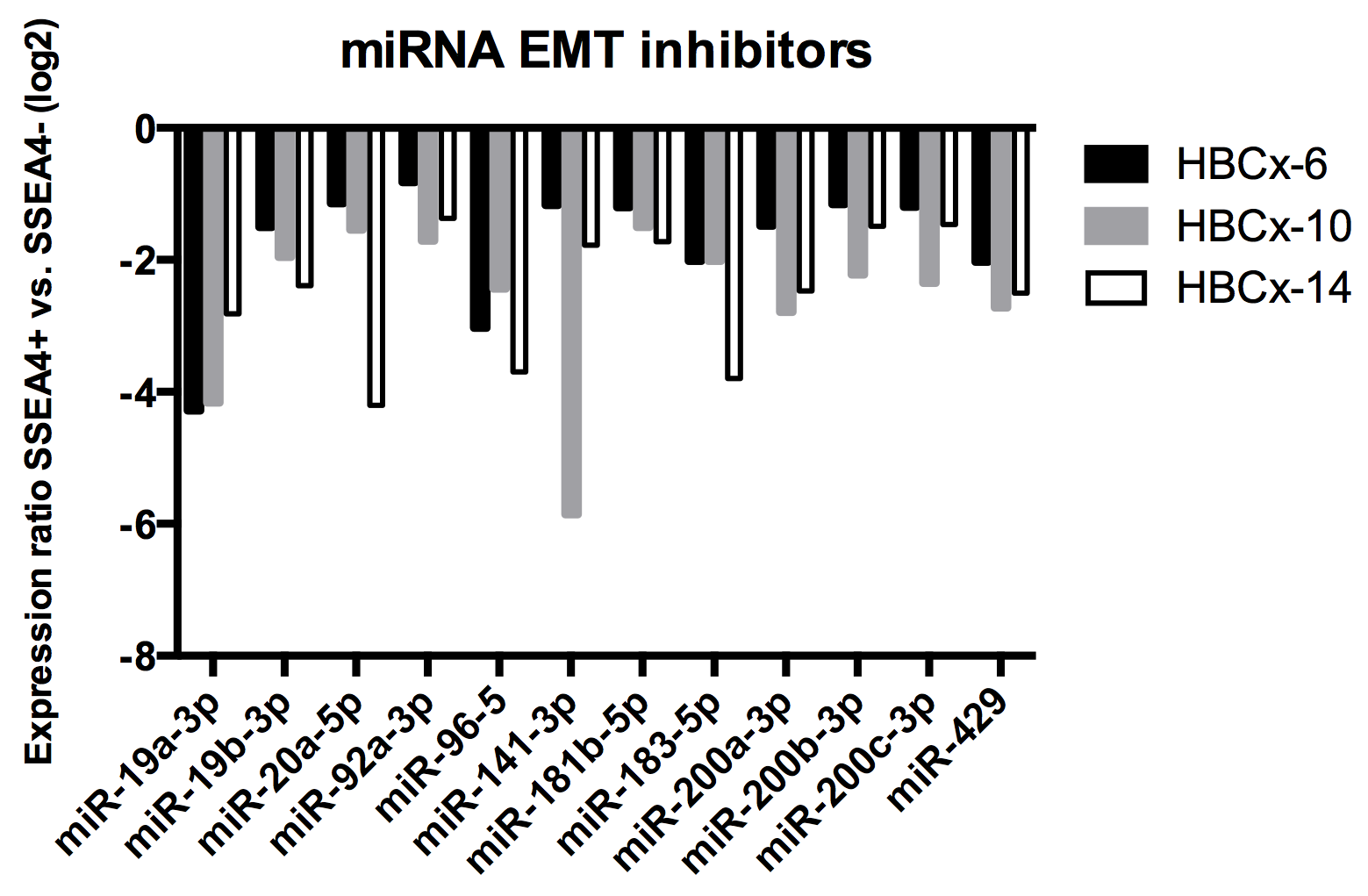

Supplement: Additional file 12: Figure S8. — SSEA4-positive breast cancer cells show decreased expression of miRNAs inhibiting EMT inducers. Expression ratios of the 12 miRNAs targeting the key mesenchymal regulator and indicator genes ZEB1, ZEB2, fibronectin 1, Snail1, Snail2, and Twist. Each bar represents the log2 expression ratio of the SSEA4-positive fraction relative to the SSEA4-negative fraction for the respective tumor model. (TIFF 201 kb) [file 13058_2015_652_MOESM12_ESM.tiff]

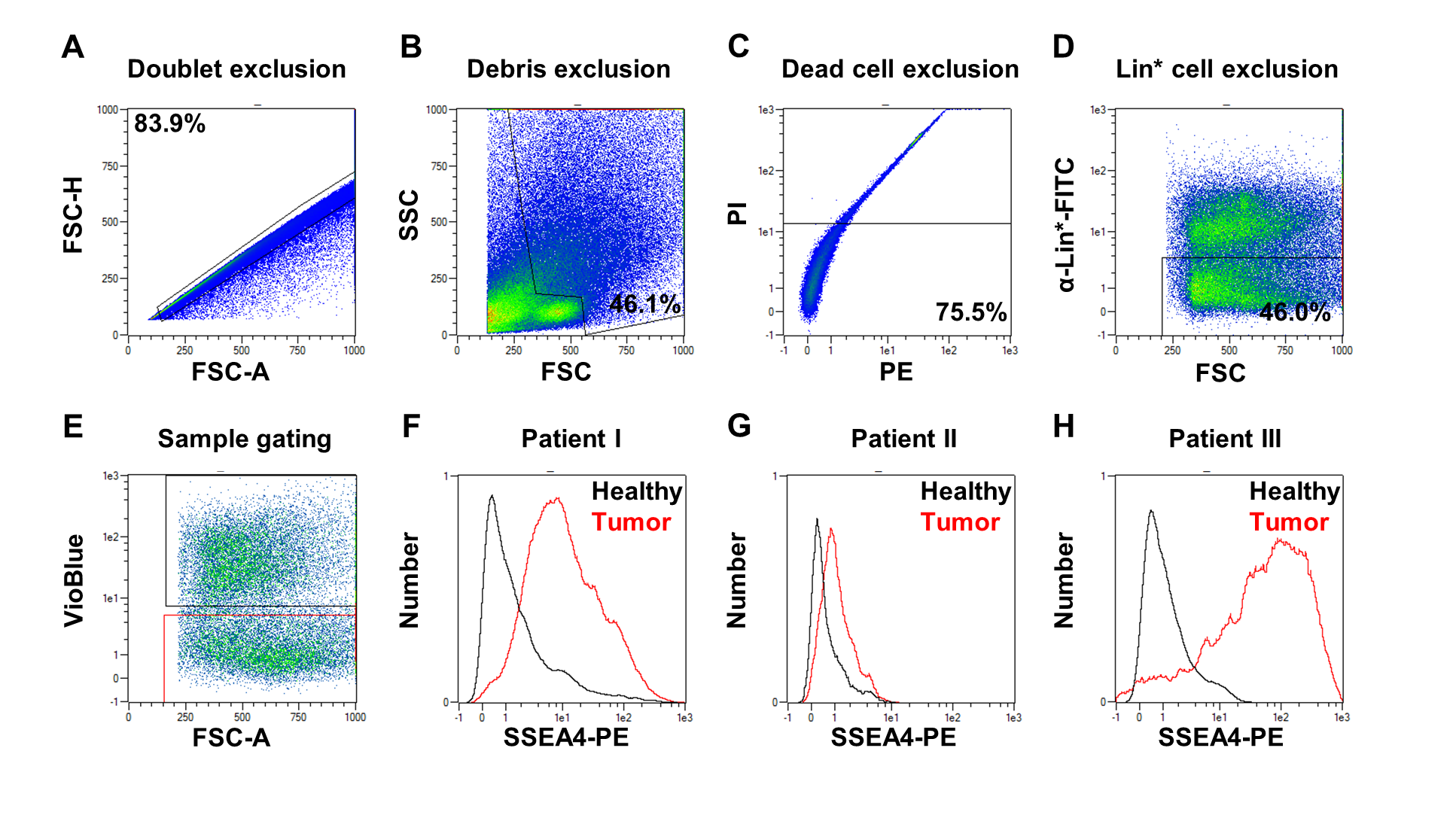

Supplement: Additional file 13: Figure S9. — Expression of SSEA4 in RCC and healthy kidney tissue. Primary RCC and healthy kidney tissues from the same patient were dissociated and analyzed by multiparametric flow cytometry. Doublets were excluded by FSC-A/FSC-H gating (a); debris was excluded by FSC/SSC gating (b); dead cells were excluded by gating off PI+ events (c); and lineage-positive cells were excluded by gating on α-Lin-FITC–negative events (d). In each patient, healthy and tumor tissues were analyzed in parallel in one labeling reaction. Therefore, one of the samples was labeled using a UV dye, allowing for subsequent separation of the events of each sample by gating on the VioBlue channel fluorescence intensity (e–h). In all of the analyzed patients (n = 3), the expression of SSEA4 was strongly increased in the tumor tissue as compared with the respective healthy tissue, with almost all tumor cells expressing SSEA4 in two of the patients (f–h). *α-Lin-FITC = CD45-FITC, CD31-FITC, CD235a (glycophorin A)-FITC. (PNG 717 kb) [file 13058_2015_652_MOESM13_ESM.png]
